# *S*-Adenosyl-L-Homocysteine Hydrolase Inhibition by a Synthetic Nicotinamide Cofactor Biomimetic

**DOI:** 10.3389/fmicb.2018.00505

**Published:** 2018-03-21

**Authors:** Lyn L. Kailing, Daniela Bertinetti, Caroline E. Paul, Tomasz Manszewski, Mariusz Jaskolski, Friedrich W. Herberg, Ioannis V. Pavlidis

**Affiliations:** ^1^Department of Biochemistry, University of Kassel, Kassel, Germany; ^2^Laboratory of Organic Chemistry, Wageningen University & Research, Wageningen, Netherlands; ^3^Center for Biocrystallographic Research, Institute of Bioorganic Chemistry, Polish Academy of Sciences, Poznań, Poland; ^4^Department of Crystallography, Faculty of Chemistry, Adam Mickiewicz University in Poznań, Poznań, Poland; ^5^Department of Chemistry, University of Crete, Heraklion, Greece

**Keywords:** *S*-adenosyl-L-homocysteine hydrolase, inhibition, nicotinamide cofactor, biomimetic, crystallography

## Abstract

*S*-adenosyl-L-homocysteine (SAH) hydrolases (SAHases) are involved in the regulation of methylation reactions in many organisms and are thus crucial for numerous cellular functions. Consequently, their dysregulation is associated with severe health problems. The SAHase-catalyzed reaction is reversible and both directions depend on the redox activity of nicotinamide adenine dinucleotide (NAD^+^) as a cofactor. Therefore, nicotinamide cofactor biomimetics (NCB) are a promising tool to modulate SAHase activity. In the present *in vitro* study, we investigated 10 synthetic truncated NAD^+^ analogs against a SAHase from the root-nodulating bacterium *Bradyrhizobium elkanii*. Among this set of analogs, one was identified to inhibit the SAHase in both directions. Isothermal titration calorimetry (ITC) and crystallography experiments suggest that the inhibitory effect is not mediated by a direct interaction with the protein. Neither the apo-enzyme (i.e., deprived of the natural cofactor), nor the holo-enzyme (i.e., in the NAD^+^-bound state) were found to bind the inhibitor. Yet, enzyme kinetics point to a non-competitive inhibition mechanism, where the inhibitor acts on both, the enzyme and enzyme-SAH complex. Based on our experimental results, we hypothesize that the NCB inhibits the enzyme via oxidation of the enzyme-bound NADH, which may be accessible through an open molecular gate, leaving the enzyme stalled in a configuration with oxidized cofactor, where the reaction intermediate can be neither converted nor released. Since the reaction mechanism of SAHase is quite uncommon, this kind of inhibition could be a viable pharmacological route, with a low risk of off-target effects. The NCB presented in this work could be used as a template for the development of more potent SAHase inhibitors.

## Introduction

**FIGURE 1:**
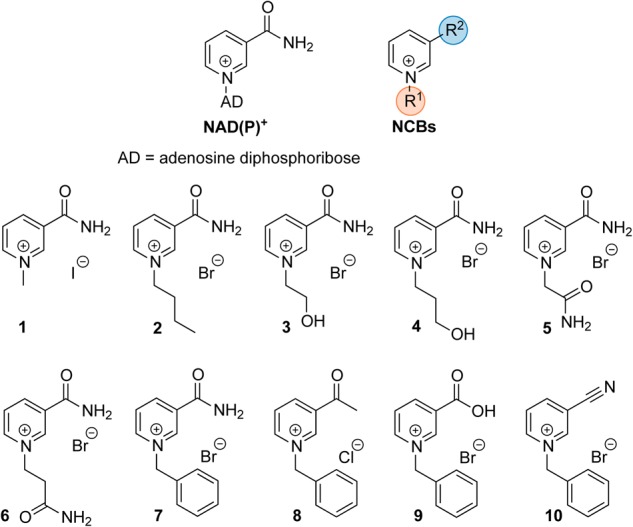
Schematic structure of the natural cofactor NAD(P)^+^ (Left) and synthetic NCBs (Right and Bottom). R^1^ and R^2^ denote the varying substituents at the pyridinium nitrogen and the C-3 position in NCB **1**–**10**.

Synthetic cofactor analogs proved their potential to replace the natural nicotinamide adenine dinucleotide (phosphate) cofactor NAD(P)H in certain oxidoreductases (Paul et al., 2013, 2014; Paul and Hollmann, 2016). Whether the analogs were used to modulate the electron transfer process (Paul et al., 2015) or the kinetics of the enzymatic reaction (Knaus et al., 2016), full comprehension of the protein-cofactor interactions requires further research. A better understanding of the molecular processes will facilitate in turn the design and preparation of tailor-made truncated synthetic biomimetics. In this study, a series of oxidized nicotinamide cofactor biomimetics (NCBs), with different substituents at the pyridinium nitrogen and C3 positions (**Figure 1**), were investigated. The NCBs display different properties with regard to their redox potential, hydrogen bonding, and hydrophobicity, which are reflected in their enzyme-dependent ability to confer activity. In a set of reduced NCBs for example, the substitution of the C3 amide moiety for a nitrile group (as in NCB **10**) was found to lead to substantially lower activity of an ene reductase, compared to the “amide” NCBs or the natural reduced nicotinamide cofactor. This was proposed to be accounted for by the hydrogen bonding of the nitrile group to only one histidine residue out of the usual two (Paul et al., 2013), a different redox potential (less negative than that of the natural cofactor), or the distance between electron donor and acceptor groups (Geddes et al., 2016). NCBs can be used in prodrug therapy (Knox et al., 2000), and hence enzyme inhibition through cofactors can play a prospectively important role in the design of novel pharmaceuticals (Giangreco and Packer, 2013).

**FIGURE 2:**
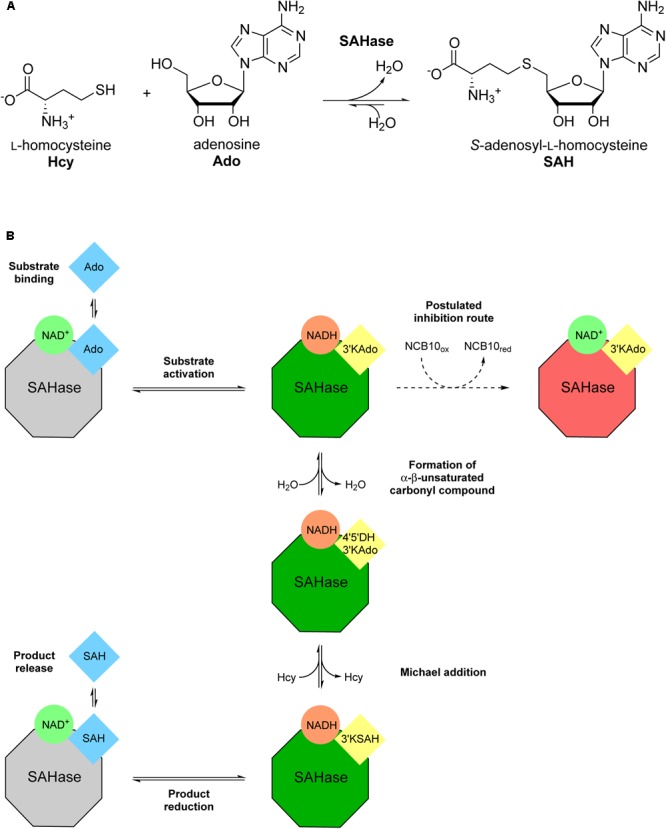
Illustration of the reversible *Bradyrhizobium elkanii* SAHase-catalyzed *S*-adenosyl-L-homocysteine (SAH) hydrolysis to adenosine (Ado) and L-homocysteine (Hcy). **(A)** Reaction scheme with chemical structures of substrates and products. **(B)** Schematic depiction of the catalytic cycle and the postulated inhibition mechanism. Color coding highlights the redox state of the cofactor (green circles: oxidized form; orange circles: reduced form) and the substrate/intermediate/product (blue squares: reduced C3′; yellow squares: oxidized C3′) as well as the state of the enzyme (gray octagons: not activated; green octagons: activated; red octagons: inhibited). Intermediate names (in yellow squares) were abbreviated as follows: 3′-ketoadenosine, 3′KAdo; 4′,5′-dehydro-3′-ketoadenosine, 4′5′DH3′KAdo; 3′keto-*S*-adenosyl-L-homocysteine, 3′KSAH. Bold annotations beside arrows describe the respective stage of the catalytic cycle in the direction of SAH synthesis.

Several semi-natural nicotinamide adenine dinucleotide analogs (i.e., modification of the nicotinamide or adenine moieties only) were previously investigated with *S*-adenosyl-L-homocysteine hydrolase (SAHase) from human sources and *Trypanosoma cruzi* (Li et al., 2009). SAHases (EC 3.3.1.1) catalyze the reversible hydrolysis of *S*-adenosyl-L-homocysteine (SAH), an important metabolite in the context of cellular methyltransferase regulation, to adenosine (Ado) and L-homocysteine (Hcy) (**Figure 2A**). SAHase is a potential pharmacological target, as abnormal levels of SAH can lead to pathological conditions, such as cardiovascular diseases (Valli et al., 2008; Xiao et al., 2015). For this reason, the regulation of their activity is of utmost importance (Li et al., 2009). Herein we present the screening of several synthetic truncated NCBs and their effect on the activity of the SAHase homolog from *Bradyrhizobium elkanii* as a model enzyme. Since one of the NCBs turned out to be an inhibitor, a thorough characterization of the interactions with the enzyme has been carried out to elucidate the inhibition mechanism.

## Materials and Methods

### Materials

N-terminally His-tagged *Bradyrhizobium elkanii* SAHase (BeSAHase) was recombinantly expressed in *E. coli* from a pET151/D-TOPO vector (Manszewski et al., 2015). If not stated otherwise, chemicals were purchased from Sigma and were of analytical grade. The NCBs **1–10** were synthesized according to the procedures adapted from previous studies (Knox et al., 2000; Paul et al., 2013), as presented in the Supplementary Material, where their full characterization is reported.

### Protein Expression and Purification

For all ITC and activity measurements, recombinant BeSAHase production was carried out as described by Kailing et al. (2017). Metal-ion affinity purification yielded holo-SAHase, since the cofactor NAD^+^ co-purifies with the enzyme. NAD^+^ removal, yielding apo-SAHase, was achieved by precipitation with ammonium sulfate, using a modified protocol of Brzezinski et al. (2008) with the protein stock diluted to a concentration of ∼1 mg/mL, as determined by the method of Bradford (1976), using bovine serum albumin as standard. Five milliliters of enzyme solution were mixed with 10 mL of acidified precipitation solution [saturated (NH_4_)_2_SO_4_ solution supplemented with 2.5 mM DTT and 1 mM EDTA, acidified to pH 3.3 with H_2_SO_4_] and incubated at 4°C on a rotator for 30 min. The suspension was centrifuged at 3,320 *× g* for 10 min. The supernatant was discarded, the precipitate was resuspended in 5 mL Tris buffer (20 mM Tris–HCl, pH 8), supplemented with 500 mM NaCl, and again mixed with 10 mL of acidified precipitation solution. Incubation and centrifugation were repeated as described above, the pellet was resuspended in 10 mL of non-acidified precipitation solution and immediately subjected to centrifugation again. The supernatant was discarded and the resulting apo-protein pellet was either resuspended in 5 mL Tris buffer and dialyzed four times against 750 mL NaMOPS buffer (20 mM MOPS, 150 mM NaCl, pH 7) to remove residual ammonium sulfate, or stored at -20°C. Frozen protein pellets with ammonium sulfate were stable for long-term storage without loss in specific activity when thawed and resuspended in buffer.

For crystallization, BeSAHase expression and purification was performed as described earlier (Manszewski et al., 2017), using a modified procedure P2, which included protein precipitation with ammonium sulfate as above to remove all ligand molecules bound by the protein at the overexpression step. The only difference to the original P2 procedure was that no NAD^+^ was added to the apo-protein solution. Instead, the solution was divided into two portions, **A** and **B**, both of which were incubated for 12 h with 12-fold molar excess of NCB **10**, while sample **B** was additionally (simultaneously) incubated with the same molar excess of adenosine (Ado).

### HPLC-Based Activity Assay for Endpoint Measurements in the Synthetic Direction

HPLC-based activity measurements in the direction of SAH synthesis were carried out as described by Kailing et al. (2017), with constant substrate concentrations (0.5 mM) of both Ado and Hcy. For the cofactor screenings, the assay was supplemented with 2 mM of the respective compound and reactions were initiated with ∼10 μg (700 nM final concentrations in assay) of apo- or holo-BeSAHase, respectively. One set of reactions was terminated after 30 min, the other after 17 h. Half-maximum inhibitory concentrations (IC_50_) of NCB **10** were measured with 100 nM NAD^+^-saturated SAHase (apo-SAHase that was pre-incubated for at least 2 h with 1 mM NAD^+^). Reactions were terminated after 30 min. Conversions were derived from HPLC chromatograms as described before (Kailing et al., 2017), plotted against the concentration of inhibitor and a linear regression analysis was performed with the GraphPad Prism software (v. 6.01). The IC_50_ was calculated as the concentration leading to half-maximum effect of the inhibitor.

### Kinetic Measurements in the Hydrolytic Direction

Reactions were performed at room temperature in 100 μL sodium phosphate buffer (100 mM, pH 8), supplemented with 1 mM EDTA, 0.2 mM 5,5′-dithiobis(2-nitrobenzoic acid) (DTNB) and SAHase. In experiments with defined NAD^+^ concentrations, apo-SAHase was pre-incubated with the respective amount of cofactor for at least 2 h, to ensure that the binding equilibrium was reached before initiation of the reaction. Enzyme concentrations were adjusted according to specific activity, to avoid exceeding maximum reaction rates (*v*_max_) of 0.01 mM⋅min^-1^. Higher reaction rates would lead to product inhibition, caused by the accumulation of Ado, in the time frame of the measurement. Reactions were initiated by addition of the substrate (SAH). The reaction rate was monitored photometrically, using DTNB to detect the formation of Hcy. Experiments were carried out in a double-beam UV-vis spectrophotometer (Specord 205, Analytik Jena). Initial reaction rates (*v*_0_) were calculated from the first 20–25 s of the reaction, based on the increase of extinction (Δ*E*) at 412 nm over time (Δ*t*) in a quartz cuvette with a path length (*d*) of 1 cm, assuming an extinction coefficient (𝜀) of 14,150 L cm^-1^ mol^-1^ for the product: v_0_ = ΔE ⋅ ${𝜀}_{\mathrm{412 nm}}^{–1}$ ⋅ d^-1^ ⋅ Δt^-1^. The assay was validated by comparison to data obtained with the coupled photometric assay described by Kailing et al. (2017). The latter assay was not used in this study, since NCB **10** contributes to oxidation of the monitoring compound NADH in a non-enzymatic way, thereby compromising the reliability of the measurement.

Determination of the IC_50_ value of NCB **10** was carried out with 285 nM NAD^+^-saturated BeSAHase (apo-SAHase after pre-incubation with 1 mM NAD^+^) and constant substrate concentrations (0.2 mM SAH). Initial reaction rates were plotted against the concentration of inhibitor and a linear regression analysis was performed as described above for the HPLC assay. The inhibitory constant (*K*_i_) was calculated from the following relation, where *K*_M_, [E] and [S] denote the Michaelis–Menten constant, concentration of enzyme and of substrate, respectively: IC_50_ = K_i_ ⋅ $K_{M}^{–1}$) + [E]_0_/2 (Cha, 1975). Characterization of the inhibition model was done by measuring Michaelis–Menten kinetics in the presence of varying inhibitor concentrations. *K*_M_ and maximum reaction rate (*v*_max_) values were obtained by non-linear Michaelis–Menten fitting using GraphPad Prism.

Kinetic-based binding studies to determine the half-maximum effective concentration (EC_50_) for NAD^+^ were carried out with 330 nM SAHase, after pre-incubation with the respective amount of NAD^+^, and constant substrate concentrations (0.2 mM SAH). Initial reaction rates were plotted against the NAD^+^ concentration and a hyperbolic fit was calculated using GraphPad Prism. The EC_50_ was calculated as the NAD^+^ concentration leading to half-maximum activation of BeSAHase.

### Isothermal Titration Calorimetry (ITC)

Interaction analyses by ITC were performed with an Affinity ITC microcalorimeter (Low Volume, 24 K Gold Cell; TA Instruments). Prior to measurements, protein was dialyzed into 20 mM MOPS buffer containing 150 mM NaCl. The buffer was adjusted to pH 7 at room temperature, but dialysis was carried out at 4°C. To ensure equal buffer conditions, ligands were dissolved and diluted in the same buffer that was used for the last step of protein dialysis. Measurements were carried out at a constant cell temperature of 25°C. The cell was equilibrated with 40–90 μM SAHase for at least 10 min before the first injection of the respective ligand. The volume of the injections was varied between 2 and 4 μL, depending on the experiment and ligand concentration. The spacing between injections was adjusted according to the time required to reach the binding equilibrium (usually 240–500 s). Ligand titration was continued until peak sizes remained constant, representing only the enthalpy of dilution. The latter was subtracted from the raw data using a mock titration of the ligand into buffer only. Data evaluation was performed with the NanoAnalyze software (v. 3.7.5; TA Instruments), using the model “independent set of multiple binding sites” (Freire et al., 1990) for *K*_D_ determination.

### Crystallization

BeSAHase solutions **A** and **B** were concentrated to 12 mg⋅mL^-1^ and used for crystallization by vapor diffusion in hanging drops at 19°C. Only solution **B** (which had been additionally incubated with Ado) gave protein crystals. The best diffracting crystals grew within 1 week in the presence of a precipitation buffer containing 0.3 M sodium acetate, 16% (w/w) PEG 400, and 0.1 M Tris pH 8.5.

### X-Ray Diffraction Data Collection, Structure Solution and Refinement

Low-temperature X-ray diffraction data were collected to 1.92 Å resolution at beamline 14.1 of the BESSY synchrotron (HZB Berlin) using a wavelength of 0.918 Å. The crystal was vitrified at 100K in a cold nitrogen gas stream after cryopreservation in the precipitation buffer supplemented with 25% (w/w) PEG 400. The crystals belong to the monoclinic system with space group *P*2_1_. The diffraction data were processed and scaled with *XDS* (Kabsch, 2010). Data collection statistics are presented in Supplementary Table S1. The structure was solved by molecular replacement with *Phaser* (McCoy et al., 2007) using chain *A* of the BeSAHase PDB entry 4lvc (Manszewski et al., 2015) as a search model. The asymmetric unit contains one complete BeSAHase tetramer. The *PHENIX* package (Adams et al., 2010) was used for the refinement of the model with maximum-likelihood targets and with TLS parameters predicted by the *TLSMD* server (Painter and Merritt, 2006). Manual model rebuilding in electron density maps was carried out in *Coot* (Emsley and Cowtan, 2004). The final structure refinement statistics are shown in Supplementary Table S1. The model of the crystal structure was standardized with *ACHESYM* (Kowiel et al., 2014) and the atomic coordinates and structure factors were deposited in the Protein Data Bank (PDB) with the accession code 6exi. Raw diffraction images were deposited in the RepOD repository (ICM, University of Warsaw) with the following DOI: http://dx.doi.org/10.18150/repod.1173852.

## Results

### Screening of SAHase Activity in the Presence of Synthetic NCBs

Recent studies have shown that the reduced form of certain NCBs can reconstitute activity in several oxidoreductases in the absence of the natural nicotinamide adenine dinucleotide cofactor (Paul et al., 2013, 2014; Paul and Hollmann, 2016; Nowak et al., 2017b). In this work, we sought to expand the screen to a different class of cofactor-requiring enzymes, the NAD^+^-dependent SAHases (EC 3.3.1.1), due to their interesting role as a pharmacological target. The initial screen was performed with a set of 10 NCBs (presented in **Figure 1**) on apo- as well as holo-enzyme. While none of the selected NCBs could reconstitute activity of apo-SAHase (even after extended incubation times of 17 h), re-supplementation with NAD^+^ recovered highly active enzyme (full substrate conversion in less than 30 min under conditions described for the HPLC-based assay). However, a screen of the NCBs with holo-SAHase (in the presence of excess NAD^+^) revealed that NCB **10** led to significantly lower substrate conversion (∼75% inhibition with 2 mM of **10**), whereas the other analogs did not affect the enzymatic activity.

### Binding of Natural Cofactor to Apo-SAHase

**FIGURE 3:**
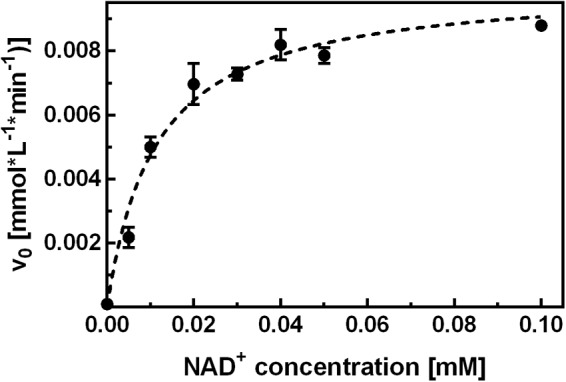
Kinetic characterization of NAD^+^ binding. Apo-SAHase was pre-incubated with varying NAD^+^ concentrations and activity was measured in the hydrolytic direction. Hyperbolic fitting (dashed curve) yielded an EC_50_ of 11.2 ± 1.4 μM (*r*^2^ = 0.959) for NAD^+^. Displayed are average values and standard deviations of replicates (*n* = 3).

To investigate the inhibition mechanism of NCB **10**, it was first necessary to characterize the binding and function of the natural cofactor. To this end, binding studies were performed by means of ITC and the hydrolytic activity assay. In separate ITC experiments, the apo-enzyme was titrated with NAD^+^ or NADH. For both cofactors binding was enthalpy-driven, with an unfavorable entropy change, and a relatively slow return to the equilibrium state was observed upon cofactor injection. Fitting of the isotherms yielded *K*_D_ values in the low micromolar range. To take account of the slow cofactor association, all subsequent activity measurements were performed after a minimum 2 h pre-incubation of apo-enzyme with the respective NAD^+^ concentration. Control experiments showed that adding NAD^+^ only immediately before initiation of the reaction was not sufficient to elevate the hydrolytic activity above background levels within 5 min measuring time. Another approach to investigate the affinity of NAD^+^ for apo-SAHase was performed according to an experiment described by Li M. et al., 2007, where the activity of SAHase was measured as a function of the NAD^+^ concentration provided for pre-incubation. Our experimental data yielded an EC_50_ value of ∼10 μM (**Figure 3**).

### Inhibition by NCB **10**

**FIGURE 4:**
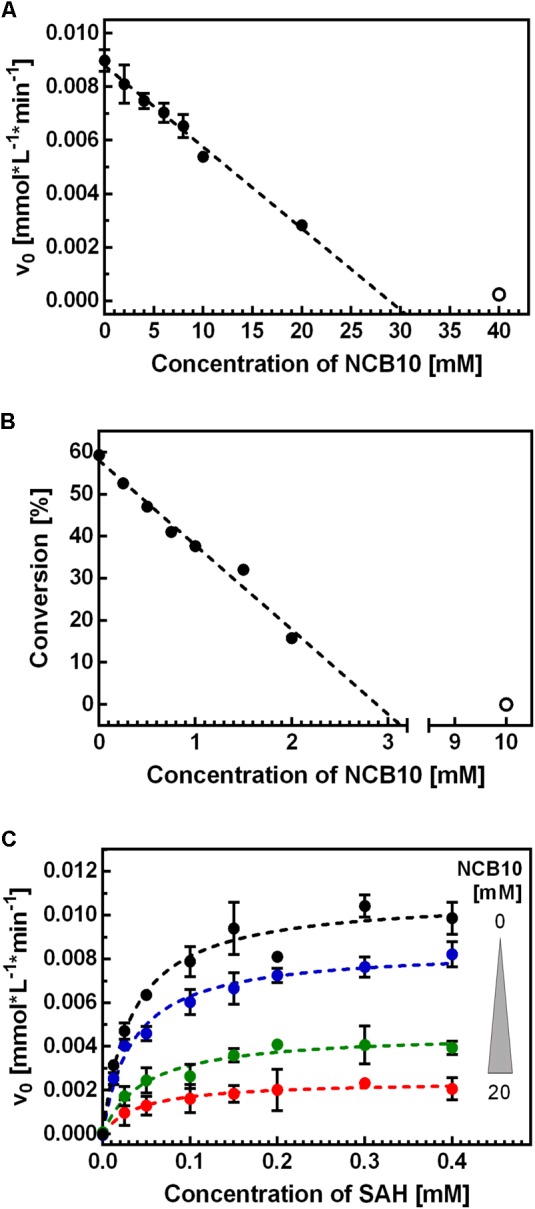
Characterization of the inhibitory effect of NCB **10** on BeSAHase. The IC_50_ of **10** was determined in the hydrolytic **(A)** and synthetic **(B)** direction. Only solid circles were considered for linear regression (dashed lines) which yielded IC_50_ values of 14.4 ± 0.5 mM (*r*^2^ = 0.969) and 1.44 ± 0.04 mM (*r*^2^ = 0.974) for hydrolysis and synthesis, respectively. The hollow circles were excluded from the analysis, due to loss of linear relation in this range, but are displayed as an example of complete inhibition. **(C)** Michaelis–Menten kinetics of SAH hydrolysis were carried out with varying concentrations of **10**. Color-coding corresponds to the inhibitor concentration (black: 0 mM; blue: 5 mM; green: 15 mM; red: 20 mM). Dashed lines represent the Michaels–Menten fits. Displayed are average values and standard deviations of replicates (**A**: *n* = 2; **B,C**: *n* = 3).

Inhibition tests with holo-BeSAHase showed a clear concentration dependence on NCB **10** (**Figure 4**). Interestingly, the IC_50_ differed by a factor of 10 between the two directions of the enzymatic reaction, with ∼14 mM for hydrolysis (**Figure 4A**) and ∼1.4 mM for synthesis (**Figure 4B**). However, we cannot conclude from these data that the inhibition mechanism is direction-sensitive, because the IC_50_ value depends on enzyme and substrate concentration, as well as on the *K*_M_ value. For the hydrolytic direction these parameters were available, thus we could determine the inhibitory constant *K*_i,hydrolysis_ as ∼2 mM. However, Michaelis–Menten kinetics for the synthetic direction are challenging, as the activity is determined using endpoint kinetics. To learn more about the inhibition mechanism, we measured full Michaelis–Menten kinetics for the hydrolytic direction as a function of varying inhibitor concentrations (**Figure 4C**). Supplementary Table S2 presents the resulting *K*_M_ and *v*_max_ values for each inhibitor concentration. Since the *K*_M_ values remained constant but *v*_max_ decreased with rising inhibitor concentrations, we concluded that the inhibition follows a non-competitive mechanism.

Apart from its effect on enzymatic activity, we also investigated NCB **10** in terms of binding to BeSAHase (by ITC). Titration of apo- as well as holo-BeSAHase with **10** resulted in small binding enthalpies, indicating either an entropy-driven binding mode, or no direct interaction with the enzyme. To exclude any interaction or chemical reaction with the reactants, we performed a simple co-incubation experiment of **10** with all reaction components (Ado, Hcy, SAH, NAD^+^ and the intermediate cofactor state NADH) in separate aliquots. Samples were subjected to HPLC analysis and examined for altered peaks. Only incubation with NADH revealed an effect: a new peak emerged at the characteristic retention time of NAD^+^, which showed no absorbance at 340 nm. These observations made us conclude that NADH was oxidized to NAD^+^. A control sample of NADH that has been incubated under similar conditions over the same period, but without **10**, confirmed that the effect was not caused by auto-oxidation.

### Crystallization Experiments of BeSAHase in the Presence of NCB **10**

**FIGURE 5:**
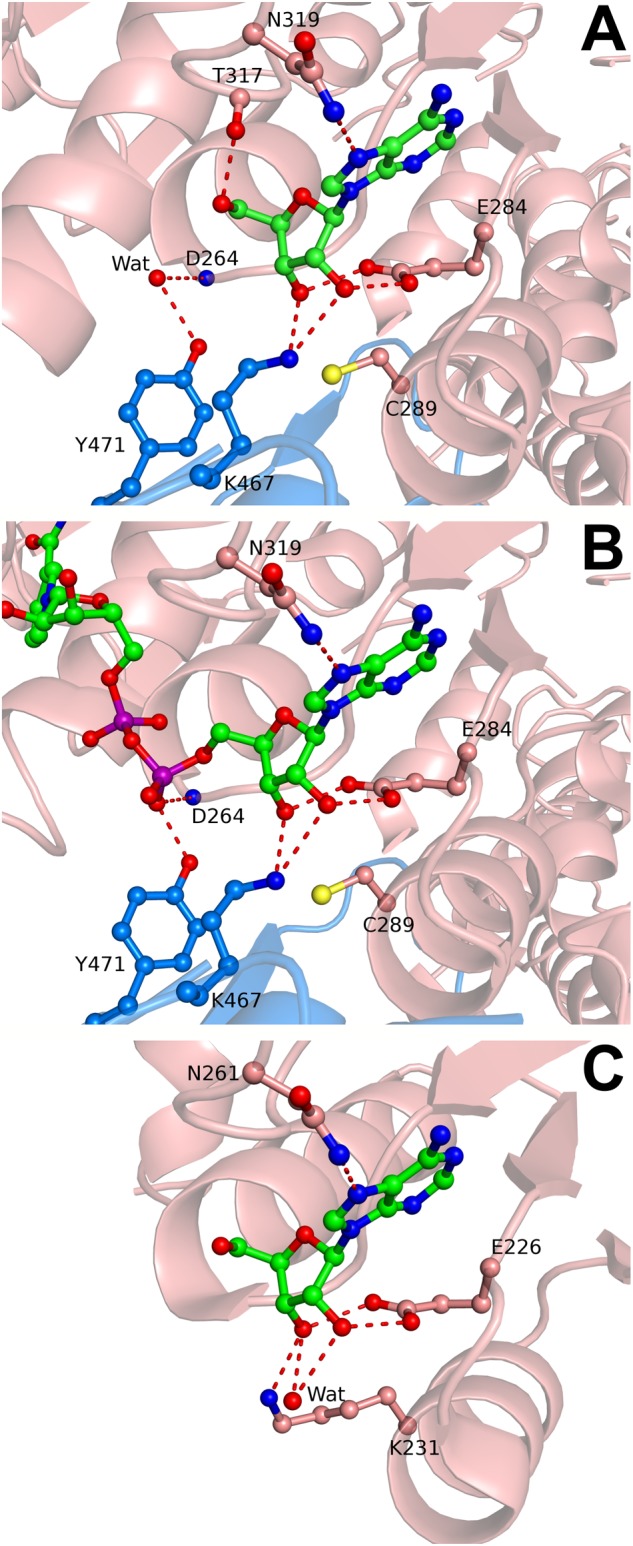
Adenosine and NAD^+^ binding in the cofactor site of SAHase crystal structures. **(A)** Ado2 (ball-and-stick) bound in the cofactor binding site of subunit A (salmon) of BeSAHase from the present study, shown in mF_o_-DF_c_ difference OMIT electron density map contoured at 3σ. Hydrogen bonds are represented by dashed lines. A water molecule that mimics a P-O^-^ oxygen atom of NAD^+^ is shown as a red sphere and denoted as Wat. **(B)** NAD^+^ molecule (ball-and-stick) bound in subunit A of BeSAHase PDB model 5m66 (Manszewski et al., 2017). Colors/hydrogen bonds as in **A**. **(C)** Ado2 (ball-and-stick) bound in the cofactor binding site of TmSAHase (PDB entry 5tow; Brzezinski et al., 2017). Lys231, equivalent of Cys289 in the BeSAHase structure, is shown in ball-and-stick representation. Water molecule/colors/hydrogen bonds as in **A**.

BeSAHase crystallized only when **10** and Ado were present simultaneously, whereas the protein incubated with **10** only did not form any crystals. The crystal belongs to the monoclinic space group *P*2_1_, which is at variance with the orthorhombic system and *P*2_1_2_1_2 space group that are characteristic of all previously determined BeSAHase structures (Manszewski et al., 2015, 2017). The present structure reveals a tetrameric assembly, which can be considered as a dimer of intimate dimers (AB and CD), even though the enzyme was deprived of NAD^+^ (because of the prior cofactor removal procedure). Indeed, the fact that no residual amounts of NAD^+^ were found in the electron density map confirmed the efficiency of the cofactor removal procedure with ammonium sulfate treatment. Although the molar excess over apo-enzyme was the same (12-fold) for both compounds (**10** and Ado), only Ado molecules were found in the electron density maps. Interestingly, each BeSAHase subunit accommodated two Ado molecules. One is located in the typical Ado binding site in the substrate-binding domain (hereafter referred to as Ado1; **Figure 5A**), forming the same hydrogen bonding network as in the previously reported structures. The other site (hereafter referred to as Ado2) coincides with the position that was previously shown to be occupied by the adenosine moiety of the NAD^+^ cofactor in the holo-enzyme (**Figure 5B**; Manszewski et al., 2015, 2017). Only the O5′ atom of Ado2 could not be aligned with any of the atoms of the NAD^+^ template. Otherwise, Ado2 is coordinated by Lys467 of the neighboring subunit (which uses its N^ζ^ atom to form hydrogen bonds with O2′ and O3′ of the ribose moiety, with average bond lengths of 2.99 and 2.73 Å, respectively) in the same way as seen for the equivalent moiety of NAD^+^. Another similarity in hydrogen bonding, regarding the nucleotide/Ado2 binding pocket, is found for residues Tyr471 (belonging, as Lys467, to the neighboring subunit) and Asp264 (from the parent subunit): while in the NAD^+^-bound structure these residues interact with a dinucleotide phosphate O atom (via their O^η^ and N atoms, respectively), in the present NAD^+^-free structure these interactions involve a water molecule. It is interesting to note that most of the other missing hydrogen bonding atoms of NAD^+^ are not mimicked by water molecules, unlike in several other SAHase structures (e.g., PDB entry 3one; Brzezinski et al., 2012) where an incomplete product molecule is marked by water sites.

All four subunits of the present BeSAHase tetramer assume a very similar conformation, with root-mean-square deviations (RMSD) for their C^α^ superpositions in the range of 0.23 Å (for subunits A and C) to 0.59 Å (for subunits B and D). As reported by Manszewski et al. (2017), BeSAHase can adopt three different conformations, closed, open, and semi-open, which are defined by the relative orientation of the two major domains, the substrate-binding domain (where the Ado1 site is located) and the cofactor-binding domain (in the present structure marked by Ado2). The RMSD values for C^α^ superpositions of those three conformations are 2.32 Å (closed/open), 1.71 Å (closed/semi-open), and 1.29 Å (open/semi-open). By comparison to these reference values, the four subunits of the present BeSAHase structure are definitely in the closed conformation, as the mean C^α^ RMSD values are, respectively, 0.51, 2.16, and 1.40 Å, for superpositions on the closed, open, and semi-open targets taken from subunit A of 5m66, subunit D of 4lvc, and subunit D of 5m5k.

**FIGURE 6:**
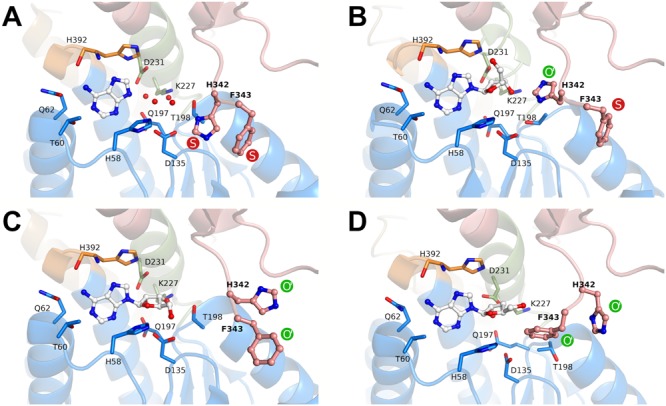
Rotamers of the molecular gate residues His342-Phe343 in different states according to crystal structures of BeSAHase. The states are denoted as shut (S, red) and open (O, green). O′ represents variants of the first reported open state (PDB entry 4lvc; Manszewski et al., 2015). **(A)** In the PDB entry 5m65 (Manszewski et al., 2017), both side chains are in the shut state. **(B)** In the present structure (PDB entry 6exi) Phe343 is in the shut state, whereas His342 assumes a formerly unknown orientation of the open state. **(C)** In subunit A of the PDB entry 5m5k (Manszewski et al., 2017), both side chains are in variant orientations of the open state. **(D)** Further variants of open state orientations (subunit D of PDB entry 5m5k; Manszewski et al., 2017). The ligand molecules in the binding site are shown in ball-and-stick representation and residues involved in ligand binding are shown as sticks. The red spheres represent water molecules. The domains are color-coded as follows: blue, substrate-binding domain; salmon, cofactor-binding domain; green, Asn222–Leu233 and orange, His392–Val396 (interdomain hinge regions).

Apart from the relative positions of the substrate- and cofactor-binding domains (here referred to as *conformation*), BeSAHase has another structural determinant, a molecular gate that controls access to a channel leading to the active site. To avoid confusion with the open and closed *conformation*, in the following text we will use the term *state* for the open (O) or shut (S) status of the molecular gate, because these two structural features do not necessarily correlate (Manszewski et al., 2017). While in the SAHase from *M. tuberculosis*, the gate was (erroneously) reported to consist of only one amino acid residue (the side chain of His363; Reddy et al., 2008), in reality it consists of a tandem of aromatic residues, which in BeSAHase have the sequence His342-Phe343 (Manszewski et al., 2015). For both residues, there is only one possible S state orientation reported (**Figure 6A**). The O state, in contrast, has been observed in several variants (Manszewski et al., 2017), with two possible orientations for His342 and three for Phe343, so far. The state of the two gating residues is not necessarily coupled: one side chain can assume the S state, while the other is in the O state, and vice versa. This is also the case in the BeSAHase structure reported here. The Phe343 side chains of all four subunits are in (the only known orientation of) the S state (**Figures 6A,B**). The side chains of His342, however, adopt an orientation of the O state that has not been observed before (**Figure 6B**). Thus, together with the previously reported orientations, the present results allow us to postulate as many as three orientations for the open state of the His342 side chain (**Figures 6B–D**).

## Discussion

### Binding of the Natural Cofactors

We have presented two different approaches in this work, to study the binding of NAD^+^ to the apo-enzyme. Although both techniques, ITC as well as the enzymatic kinetic assay, pointed to a *K*_D_ in the low micromolar range, this relatively low affinity seems counterintuitive when confronted with our observations that NAD^+^ co-purifies with the enzyme and that the interaction can even sustain extensive dialysis procedures. It is possible that NAD^+^ uptake is a cooperative process, i.e., that the affinity for the other subunits could be altered upon association with the first subunit of the tetramer. Such a process would distort the *K*_D_ determination in the titration experiment used in this work.

Another interesting observation is the slow association rate of NAD^+^, something that has been already described for other SAHase homologs, such as the ones from *Trypanosoma cruzi* or humans (Li Q.-S. et al., 2007). However, a deeper characterization of NAD^+^ association and dissociation rates resulted in a complex binding model, where two different classes of binding sites were postulated: one class was reported to bind relatively fast but weakly, and the other slowly, but with high affinity. It is possible that this model also applies to BeSAHase.

Li Q.-S. et al., 2007 also described affinity determinations for the reduced form of the natural nicotinamide adenine dinucleotide cofactor, reporting them to be in the nanomolar *K*_D_ range for both SAHases. Interestingly, we could not confirm this high affinity for NADH with BeSAHase, but found it to be in the low micromolar range. Titration of apo-BeSAHase with NADH resembled the results obtained with NAD^+^ as the titrant. However, as described above at the example of NAD^+^ binding, further experiments are necessary to investigate potential cooperativity effects and/or the existence of different classes of the cofactor binding site.

### NCBs as Potential Surrogates for NAD^+^

None of the 10 truncated NCBs investigated in the present study could substitute the natural cofactor NAD^+^ in BeSAHase to restore catalytic activity, although several enzymes have been reported before to efficiently use one or more of the NCBs **1, 2**, and **7**–**10** (in the reduced form) as a surrogate for their natural cofactor NADH and/or NADPH (Paul et al., 2013, 2014; Knaus et al., 2016; Nowak et al., 2017a,b). However, some differences should be pointed out in comparison to SAHases in general. All enzymes in the above-mentioned studies belong to the class of oxidoreductases (EC 1), whereas SAHases are hydrolases (EC 3), i.e., even the reaction types are completely unrelated. Oxidoreductases cannot regenerate the reduced or oxidized form of the nicotinamide and depend on an external supply or regeneration system. In SAHases, in contrast, the cofactor (in its oxidized form) is regenerated during the catalytic cycle.

Structural comparison of different enzymes from the oxidoreductase class inspired the idea that a common structural motif might be the determinant for whether an enzyme can accept the presented NCBs, in lieu of NAD(P)H, or not. All known enzymes that exhibit equal or even higher activities when using an NCB [compared to NAD(P)H usage], have a TIM barrel structure around their substrate/cofactor binding pocket (Scholtissek et al., 2017), whereas enzymes with Rossmann fold-like substrate sites (such as BeSAHases) did not accept the NCBs or display only minimal activity (Nowak et al., 2017b). In view of the recently published results on a Rossmann fold-containing enzyme, glucose dehydrogenase (Nowak et al., 2017b), it would be interesting to investigate how this motif is limiting the use of NCBs and whether the production of variants would allow significantly higher activity. Further experimental work to address these questions is in progress.

As already mentioned, BeSAHase fluctuates between open, semi-open, and closed conformations. C^α^ superposition of these conformations yield RMSD values of ∼2 Å (Manszewski et al., 2015, 2017). It is possible that the relatively short substituent at the pyridinium N atom of the NCBs is insufficient to establish anchoring contact points to keep it in position during the large-amplitude conformational changes of the BeSAHase catalytic cycle. For future studies, we will design NCBs with longer substituents that are functionalized with polar groups in order to mimic the missing adenosine moiety of the dinucleotide.

### The Crystal Structure

To elucidate the inhibition mechanism of NCB **10**, we also investigated its structural impact on BeSAHase. However, in the absence of any additional ligand the protein did not crystallize. We cannot categorically exclude the possibility that crystal formation of BeSAHase in complex with only **10** is merely a matter of time and/or optimization of the crystallization conditions. Nevertheless, a more likely interpretation is that the failure of the crystallization experiment rather reflects the lack of a structurally robust complex of sufficient stability to compete with ligands such as Ado. The new observation of conformationally competent tetramer formation in the absence of cofactor deserves special emphasis in this context, as it has been postulated recently that NAD^+^ may be crucial for the intimate dimer formation of SAHases and therefore also play a major structural role in the tetrameric assembly (Manszewski et al., 2015). Herein, we present evidence that the presence of Ado is sufficient for tetramer formation. The additional Ado molecule, Ado2, found in the cofactor binding site, forms hydrogen-bond interactions with Lys467 of the complementary subunit of the dimer in exactly the same way as the genuine NAD^+^ cofactor. The fact that BeSAHase would not crystallize when Ado was not provided may suggest that the interactions with the Ado moiety of NAD^+^ are indeed critical for the formation of a stable quaternary structure. However, it remains to be investigated whether the ligand in the cofactor binding pocket could be truncated even further, while still retaining the potential to stabilize the oligomerization. Based on the observation of the role of Ado2 in the present crystal structure, we can hypothesize that the lack of enzymatic activity of apo-SAHase in the presence of NCB **1**–**9** is not an issue related only to the catalytic function of the cofactor, but also to the fact that in the absence of an adenosine-containing moiety there is no dimerization and therefore no functional tetramer formation.

With regard to the occurrence of Ado2 in general, a somewhat similar situation has been observed in the structure of SAHase from *Thermotoga maritima* (TmSAHase; PDB entry 5tow; Brzezinski et al., 2017), where the cofactor binding sites of two subunits, forming one intimate dimer, are also occupied by Ado molecules (**Figure 5C**), while the remaining two subunits of the tetrameric protein are occupied by the reduced cofactor (NADH). In that case, Ado seems to be even capable of successfully competing with the cofactor for the nucleotide binding site.

Another interesting feature of the presented crystal structure is the new orientation variant of the molecular gate in BeSAHase. Our structure revealed a formerly unknown orientation of His342 in the open state. With this new variant, there are three possible orientations of the His element, suggesting that there is a high degree of flexibility that could facilitate access of the active site even during the catalytic cycle, i.e., when the protein is in the closed conformation.

### The Inhibitory Effect of NCB **10** on BeSAHase

After we found out about the inhibitory effect of NCB **10** on BeSAHase, we set out for a comprehensive characterization of this new inhibitor in order to shed more light on its mechanism. The first idea, that **10** might hamper SAHase activity by competing with NAD^+^ for the cofactor binding site, could not be confirmed in the ITC experiments. Neither titration of holo-, nor of apo-SAHase gave any hint of a binding event. This conclusion was further supported by the crystallographic results: while the adenosine part of the absent NAD^+^ cofactor was mimicked by Ado2, the nicotinamide binding area of the cofactor binding pocket remained empty, although, in theory, **10** would still fit in without steric clashes. Moreover, **10** was not even found anywhere else in the crystal structure. Together with the ITC data, this pointed to an inhibition mode which is not mediated by direct binding to the enzyme structure, but via an indirect mechanism. In parallel, we discovered that **10** oxidizes NADH in co-incubation experiments without the enzyme. Comparison of the redox potentials of both compounds at a physiological pH (NAD^+^/NADH: -320 mV; Burton and Wilson, 1953; **10_ox_**/**10_red_**: -220 mV; Ostovic et al., 1985) also confirms that this reaction can proceed spontaneously (i.e., from the thermodynamic point of view). However, initiation of the SAHase reaction relies on the oxidized form, NAD^+^, which does not react with **10**. NADH is only an intermediate and NAD^+^ is regenerated within the catalytic cycle. Indeed, we could not detect any NADH produced or released during the reaction with our analytics. Yet it is possible that the inhibitor acts on an enzyme-intermediate complex, where the cofactor is transiently in the reduced state. Formation of this intermediate configuration would theoretically only require the first step of the catalytic mechanism, the ribose C3′ oxidation of the substrate (SAH or Ado, depending on the direction; **Figure 2B**). Subsequent oxidation of enzyme-bound NADH by **10** would interrupt the catalytic cycle because the intermediate could be converted neither to the final product nor to initial substrate, since this would require the electron donating function of NADH. We hypothesize that the recovery of the active enzyme, by a simple release of the intermediate and uptake of a new substrate molecule is prevented because of high affinities of the intermediates. This hypothesis is supported by *K*_D_ determinations for bovine liver SAHase: while Ado was reported with a *K*_D_ of 9 μM, the *K*_D_ values of the intermediates 3′-ketoadenosine and 4′,5′-dehydro-3′-ketoadenosine were estimated as 600 and 300 pM, respectively (Porter and Boyd, 1992). Since high affinities of transition states are common in enzymatic catalysis, it is likely that a similar phenomenon is at work with BeSAHase.

While this postulated inhibition mechanism implies an uncompetitive mode, since the inhibitor is assumed to act only on the enzyme-intermediate complex, the presented kinetic examination, in contrast, indicates a non-competitive mechanism. The latter means, that **10** also interacts with the enzyme alone, something that we could, however, not confirm on the basis of our current experimental data. Maybe this discrepancy can be explained by the hypothesis that **10** is not a *classical* enzyme inhibitor in terms of recognizing and binding a certain structural epitope of the target protein. Nevertheless, we cannot fully exclude that **10** also has an allosteric effect on the enzyme (potentially entropy driven), in addition to its oxidizing effect on the cofactor, which might additionally contribute to the reduction of the enzymatic activity.

Another open question is how the inhibitor can enter the active site, when the catalytic cycle is already in progress, meaning that the enzyme is in the closed conformation. We suggest, that the molecular gate residues (His342 and Phe343) could provide access for the inhibitor, since it was shown in this and in previous studies that their state (open vs. shut) is not coupled with the overall subunit conformation (open vs. semi-open vs. closed). Moreover, we discovered another, so far unknown, open state configuration, which indicates that there might be even more uncharted possibilities. Observation of several orientations of a given side chain in a number of crystal structures is an unmistakable hallmark of a significant degree of dynamics in this position. Therefore, we could speculate that the open state is probably only transiently but sufficiently often available to allow access of the inhibitor.

## Conclusion

We have demonstrated that NCB **10** is a promising new inhibitor of BeSAHase and possibly of other SAHases. Though the *K*_i_ is in the millimolar range, this NCB could be used as a template in the development of more potent SAHase inhibitors, e.g., for pharmacological purposes. In contrast to previous work (Li et al., 2009), we could show that full inhibition can be induced with truncated synthetic NCBs, thus expanding the toolbox of potential inhibitors for SAHases.

## Author Contributions

LK and IP conceived and initiated the project. CP designed, synthesized, and characterized the NCBs. LK and DB designed and performed the enzymatic activity and ITC experiments. TM and MJ designed and performed the experiments on the crystallization of the protein and the analogs, and carried out the crystal structure determination and refinement. LK, DB, CP, TM, MJ, FH, and IP contributed to data interpretation and manuscript preparation. All authors have given their approval of the final version of the manuscript.

## Conflict of Interest Statement

The authors declare that the research was conducted in the absence of any commercial or financial relationships that could be construed as a potential conflict of interest.

## References

[B1] Adams P. D. Afonine P. V. Bunkóczi G. Chen V. B. Davis I. W. Echols N. (2010). PHENIX: a comprehensive Python-based system for macromolecular structure solution. *Acta Crystallogr. D Biol. Crystallogr.* 66 213–221. 10.1107/S0907444909052925 20124702 PMC2815670

[B2] Bradford M. M. (1976). A rapid and sensitive method for the quantitation of microgram quantities of protein utilizing the principle of protein-dye binding. *Anal. Biochem.* 72 248–254. 10.1016/0003-2697(76)90527-90523 942051

[B3] Brzezinski K. Bujacz G. Jaskolski M. (2008). Purification, crystallization and preliminary crystallographic studies of plant *S*-adenosyl-L-homocysteine hydrolase (*Lupinus luteus*). *Acta Crystallogr. Sect. F Struct. Biol. Cryst. Commun.* 64 671–673. 10.1107/s1744309108017703 18607106 PMC2443962

[B4] Brzezinski K. Czyrko J. Sliwiak J. Nalewajko-Sieliwoniuk E. Jaskolski M. Nocek B. (2017). *S*-adenosyl-L-homocysteine hydrolase from a hyperthermophile (*Thermotoga maritima*) is expressed in *Escherichia coli* in inactive form - Biochemical and structural studies. *Int. J. Biol. Macromol.* 104(Pt A) 584–596. 10.1016/j.ijbiomac.2017.06.065 28629859 PMC7888557

[B5] Brzezinski K. Dauter Z. Jaskolski M. (2012). High-resolution structures of complexes of plant *S*-adenosyl-L-homocysteine hydrolase (*Lupinus luteus*). *Acta Crystallogr. D* 68 218–231. 10.1107/S0907444911055090 22349223 PMC3282620

[B6] Burton K. Wilson T. H. (1953). The free-energy changes for the reduction of diphosphopyridine nucleotide and the dehydrogenation of L-malate and L-glycerol 1-phosphate. *Biochem. J.* 54 86–94. 10.1042/bj0540086 13058837 PMC1268851

[B7] Cha S. (1975). Tight-binding inhibitors—I: kinetic behavior. *Biochem. Pharmacol.* 24 2177–2185. 10.1016/0006-2952(75)90050-90057 1212266

[B8] Emsley P. Cowtan K. (2004). Coot: model-building tools for molecular graphics. *Acta Crystallogr. D* 60 2126–2132. 10.1107/S0907444904019158 15572765

[B9] Freire E. Mayorga O. L. Straume M. (1990). Isothermal titration calorimetry. *Anal. Chem.* 62 950A–959A. 10.1021/ac00217a002

[B10] Geddes A. Paul C. E. Hay S. Hollmann F. Scrutton N. S. (2016). Donor-acceptor distance sampling enhances the performance of “Better than Nature” nicotinamide coenzyme biomimetics. *J. Am. Chem. Soc.* 138 11089–11092. 10.1021/jacs.6b05625 27552302

[B11] Giangreco I. Packer M. J. (2013). Pharmacophore binding motifs for nicotinamide adenine dinucleotide analogues across multiple protein families: a detailed contact-based analysis of the interaction between proteins and NAD(P) cofactors. *J. Med. Chem.* 56 6175–6189. 10.1021/jm400644z 23889609

[B12] Kabsch W. (2010). XDS. *Acta Crystallogr. D* 66 125–132. 10.1107/S0907444909047337 20124692 PMC2815665

[B13] Kailing L. L. Bertinetti D. Herberg F. W. Pavlidis I. V. (2017). A coupled photometric assay for characterization of *S*-adenosyl-L-homocysteine hydrolases in the physiological hydrolytic direction. *N. Biotechnol.* 39 11–17. 10.1016/j.nbt.2017.04.005 28461153

[B14] Knaus T. Paul C. E. Levy C. W. De Vries S. Mutti F. G. Hollmann F. (2016). Better than nature: nicotinamide biomimetics that outperform natural coenzymes. *J. Am. Chem. Soc.* 138 1033–1039. 10.1021/jacs.5b12252 26727612 PMC4731831

[B15] Knox R. J. Jenkins T. C. Hobbs S. M. Chen S. A. Melton R. G. Burke P. J. (2000). Bioactivation of 5-(Aziridin-1-yl)-2,4-dinitrobenzamide (CB 1954) by human NAD(P)H quinone oxidoreductase 2: a novel co-substrate-mediated antitumor prodrug therapy. *Cancer Res.* 60 4179–4186. 10945627

[B16] Kowiel M. Jaskolski M. Dauter Z. (2014). *ACHESYM*: an algorithm and server for standardized placement of macromolecular models in the unit cell. *Acta Crystallogr. D* 70(Pt 12) 3290–3298. 10.1107/S1399004714024572 25478846 PMC4257622

[B17] Li M. Li Y. Chen J. Wei W. Pan X. Liu J. (2007). Copper ions inhibit *S*-adenosylhomocysteine hydrolase by causing dissociation of NAD+ cofactor. *Biochemistry* 46 11451–11458. 10.1021/bi700395d 17892301

[B18] Li Q.-S. Cai S. Borchardt R. T. Fang J. Kuczera K. Middaugh C. R. (2007). Comparative kinetics of cofactor association and dissociation for the human and trypanosomal *S*-adenosylhomocysteine hydrolases. 1. Basic features of the association and dissociation processes. *Biochemistry* 46 5798–5809. 10.1021/bi700170m 17447732

[B19] Li Q. S. Cai S. M. Fang J. W. Borchardt R. T. Kuczera K. Middaugh C. R. (2009). Evaluation of NAD(H) analogues as selective inhibitors for *Trypanosoma cruzi* S-adenosylhomocysteine hydrolase. *Nucleosides Nucleotides Nucleic Acids* 28 473–484. 10.1080/15257770903044572 20183597 PMC4127997

[B20] Manszewski T. Singh K. Imiolczyk B. Jaskolski M. (2015). An enzyme captured in two conformational states: crystal structure of *S*-adenosyl-L-homocysteine hydrolase from *Bradyrhizobium elkanii*. *Acta Crystallogr. D* 71 2422–2432. 10.1107/s1399004715018659 26627650

[B21] Manszewski T. Szpotkowski K. Jaskolski M. (2017). Crystallographic and SAXS studies of *S*-adenosyl-L-homocysteine hydrolase from *Bradyrhizobium elkanii*. *IUCrJ* 4 271–282. 10.1107/S2052252517002433 28512574 PMC5414401

[B22] McCoy A. J. Grosse-Kunstleve R. W. Adams P. D. Winn M. D. Storoni L. C. Read R. J. (2007). Phaser crystallographic software. *J. Appl. Crystallogr.* 40 658–674. 10.1107/S0021889807021206 19461840 PMC2483472

[B23] Nowak C. Pick A. Csepei L.-I. Sieber V. (2017a). Characterization of biomimetic cofactors according to stability, redox potentials, and enzymatic conversion by NADH oxidase from *Lactobacillus pentosus*. *Chembiochem* 18 1944–1949. 10.1002/cbic.201700258 28752634

[B24] Nowak C. Pick A. Lommes P. Sieber V. (2017b). Enzymatic reduction of nicotinamide biomimetic cofactors using an engineered glucose dehydrogenase: providing a regeneration system for artificial cofactors. *ACS Catal.* 7 5202–5208. 10.1021/acscatal.7b00721

[B25] Ostovic D. Lee I. S. H. Roberts R. M. G. Kreevoy M. M. (1985). Hydride transfer and oxyanion addition equilibria of NAD+ analogs. *J. Org. Chem.* 50 4206–4211. 10.1021/jo00222a006

[B26] Painter J. Merritt E. A. (2006). Optimal description of a protein structure in terms of multiple groups undergoing TLS motion. *Acta Crystallogr. D* 62 439–450. 10.1107/S0907444906005270 16552146

[B27] Paul C. E. Arends I. W. C. E. Hollmann F. (2014). Is simpler better? Synthetic nicotinamide cofactor analogues for redox chemistry. *ACS Catal.* 4 788–797. 10.1021/cs4011056

[B28] Paul C. E. Gargiulo S. Opperman D. J. Lavandera I. Gotor-Fernández V. Gotor V. (2013). Mimicking nature: synthetic nicotinamide cofactors for C=C Bioreduction using enoate reductases. *Org. Lett.* 15 180–183. 10.1021/ol303240a 23256747

[B29] Paul C. E. Hollmann F. (2016). A survey of synthetic nicotinamide cofactors in enzymatic processes. *Appl. Microbiol. Biotechnol.* 100 4773–4778. 10.1007/s00253-016-7500-7501 27094184 PMC4866995

[B30] Paul C. E. Tischler D. Riedel A. Heine T. Itoh N. Hollmann F. (2015). Nonenzymatic regeneration of styrene monooxygenase for catalysis. *ACS Catal.* 5 2961–2965. 10.1021/acscatal.5b00041 12837091

[B31] Porter D. J. Boyd F. L. (1992). Reduced *S*-adenosylhomocysteine hydrolase. Kinetics and thermodynamics for binding of 3′-ketoadenosine, adenosine, and adenine. *J. Biol. Chem.* 267 3205–3213. 1737776

[B32] Reddy M. C. Kuppan G. Shetty N. D. Owen J. L. Ioerger T. R. Sacchettini J. C. (2008). Crystal structures of *Mycobacterium tuberculosis* S-adenosyl-L-homocysteine hydrolase in ternary complex with substrate and inhibitors. *Protein Sci.* 17 2134–2144. 10.1110/ps.038125.108 18815415 PMC2590921

[B33] Scholtissek A. Tischler D. Westphal A. H. van Berkel W. J. H. Paul C. E. (2017). Old yellow enzyme-catalysed asymmetric hydrogenation: linking family roots with improved catalysis. *Catalysts* 7:130 10.3390/catal7050130

[B34] Valli A. Carrero J. J. Qureshi A. R. Garibotto G. Bárány P. Axelsson J. (2008). Elevated serum levels of *S*-adenosylhomocysteine, but not homocysteine, are associated with cardiovascular disease in stage 5 chronic kidney disease patients. *Clin. Chim. Acta* 395 106–110. 10.1016/j.cca.2008.05.018 18565329

[B35] Xiao Y. Su X. Huang W. Zhang J. Peng C. Huang H. (2015). Role of S-adenosylhomocysteine in cardiovascular disease and its potential epigenetic mechanism. *Int. J. Biochem. Cell Biol.* 67 158–66. 10.1016/j.biocel.2015.06.015 26117455

