# Supplementary material for: S-Adenosyl-L-Homocysteine Hydrolase Inhibition by a Synthetic Nicotinamide Cofactor Biomimetic
Source: Front Microbiol. 2018 Mar 21;9:505. doi: 10.3389/fmicb.2018.00505 (PMC5871694; doi:10.3389/fmicb.2018.00505)
Supplement: Supplementary file 1 [file Data_Sheet_1.pdf]

Supplementary Material

S-Adenosyl-L-Homocysteine Hydrolase Inhibition by a Synthetic Nicotinamide Cofactor Biomimetic

Lyn L. Kailing[1], Daniela Bertinetti[1], Caroline E. Paul[2], Tomasz Manszewski[3], Mariusz Jaskolski[3,4], Friedrich W. Herberg[1], Ioannis V. Pavlidis[1,5*]

[1]Department of Biochemistry, University of Kassel, Kassel, Germany

[2]Laboratory of Organic Chemistry, Wageningen University & Research, Wageningen, Netherlands

[3]Center for Biocrystallographic Research, Institute of Bioorganic Chemistry, Polish Academy of Sciences, Poznań, Poland

[4]Department of Crystallography, Faculty of Chemistry, A. Mickiewicz University, Poznań, Poland

[5]Department of Chemistry, University of Crete, Heraklion, Greece

*** Correspondence:**
Dr. Ioannis V. Pavlidis
ipavlidis@uoc.gr

1. General information on synthesis of nicotinamide cofactor mimics

All commercial reagents and solvents were purchased with the highest quality available and used as received without further purification.

Nuclear magnetic resonance (NMR) spectra were recorded on an Agilent Technologies spectrometer at 400 MHz (^{1}H). Chemical shifts (δ) are reported in parts per million (ppm) relative to Me_4Si (δ 0 ppm) using the solvent residual signals of deuterated solvent (DMSO-d_6 or $CDCl_3$).

High resolution mass spectra were recorded on a Q-Exactive mass spectrometer (Thermo) equipped with an electrospray ion source in positive mode with a resolution R = 70000 at *m*/*z* 200 (mass range *m*/*z* = 100-750) and dioctylphtalate (*m*/*z* = 391.28428) as lock mass.

2. Synthesis of the nicotinamide cofactor mimics

The following compounds were previously synthesised and characterised as reported in the literature[1]: 1-butyl-3-carbamoylpyridinium bromide [CAS 63405-87-8], 1-benzyl-3-carbamoylpyridinium bromide [CAS 13076-43-2], 1-benzyl-3-acetylpyridinium chloride [CAS 16183-85-0], 1-benzyl-3-cyanopyridinium bromide [CAS 6516-53-6], 1-benzyl-3-carboxypyridinium bromide [CAS 63405-87-8], 1-(4-sulfonatobutyl)-3-carbamoylpyridinium [CAS 52047-80-0][2]. Briefly, nicotinamide and the alkylating agent were mixed in acetonitrile and gently refluxed overnight. After cooling, the precipitate was filtered, washed with diethyl ether and dried.

1-Benzyl-3-cyanopyridinium bromide [CAS 6516-53-6]

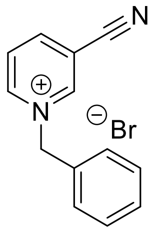

Synthesised as previously described above to afford a white powder.[1] **^{1}H NMR** (400 MHz, DMSO-d_6) δ 10.08 (d, *J* = 1.5 Hz, 1H), 9.50 (dt, *J* = 6.3, 1.3 Hz, 1H), 9.13 (dt, *J* = 8.1, 1.4 Hz, 1H), 8.38 (dd, *J* = 8.1, 6.2 Hz, 1H), 7.68 – 7.58 (m, 2H), 7.51 – 7.39 (m, 3H), 5.96 (s, 2H). **^{13}C NMR** (100 MHz, DMSO-d_6) δ 149.1, 149.0, 148.0, 133.4, 129.6, 129.3, 129.2, 128.9, 113.9, 113.2, 63.8.

The compounds below were prepared and characterized as previously described by Knox *et al.*:[2]

1-Methyl-3-carbamoylpyridinium iodide [CAS 6456-44-6]

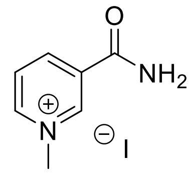

Nicotinamide (2 g, 16.4 mmol) and iodomethane (2.4 g, 16.9 mmol) were mixed in DMF (5 mL) and gently refluxed overnight. Then the reaction mixture was cooled, the precipitate was filtered, washed with diethyl ether and dried to afford a light yellow powder. **^{1}H NMR** (400 MHz, DMSO-d_6) δ 9.39 (t, *J* = 1.5 Hz, 1H), 9.13 (dt, *J* = 6.2, 1.4 Hz, 1H), 8.89 (dt, *J* = 8.2, 1.5 Hz, 1H), 8.48 (br s, 1H), 8.25 (dd, *J* = 8.1, 6.1 Hz, 1H), 8.11 (br s, 1H), 4.41 (s, 3H). **^{13}C NMR** (100 MHz, DMSO-d_6) δ 162.8, 147.1, 145.6, 142.9, 133.1, 127.4, 48.4.

1-(2-Hydroxyethyl)-3-carbamoylpyridinium bromide [CAS 51527-80-1]

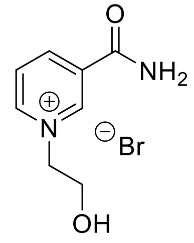

Nicotinamide (2.4 g, 20 mmol) and 2-bromoethanol (8 mL, 100 mmol) were mixed in acetonitrile (50 mL) and gently refluxed overnight. Then the reaction mixture was cooled, the precipitate was filtered, washed with diethyl ether and dried. Recrystallization from ethanol gave colorless crystals with 72% yield (3.5 g). **^{1}H NMR** (400 MHz, DMSO-d_6) δ 9.49 (d, *J* = 2.1 Hz, 1H), 9.21 – 9.14 (m, 1H), 9.04 – 8.96 (m, 1H), 8.64 (br s, 1H), 8.28 (dd, *J* = 8.1, 6.1 Hz, 1H), 8.17 (br s, 1H), 5.23 (br s, 1H), 4.75 (t, *J* = 4.9 Hz, 2H), 3.89 (dd, *J* = 5.9, 3.9 Hz, 2H). **^{13}C NMR** (100 MHz, DMSO-d_6) δ 162.9, 146.9, 145.1, 143.5, 133.4, 127.5, 63.4, 59.9. **HRMS** *m*/*z* calculated for $C_8H_{11}O_2N_2$ $[M]^+$: 167.0815, found: 167.08156.

1-(3-Hydroxypropyl)-3-carbamoylpyridinium bromide [CAS 126298-92-8]

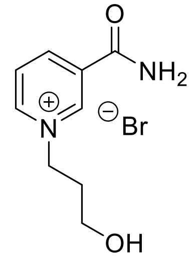

Nicotinamide (5 g, 40.9 mmol) and 3-bromo-1-propanol (4.0 mL, 45 mmol) were mixed in acetonitrile (50 mL) and refluxed overnight. Then the reaction mixture was cooled, the precipitate was filtered, washed with diethyl ether and dried to afford a white powder. **^{1}H NMR** (400 MHz, DMSO-d_6) δ 9.54 (d, *J* = 1.9 Hz, 1H), 9.25 (dd, *J* = 6.1, 1.4 Hz, 1H), 8.96 (dt, *J* = 8.1, 1.5 Hz, 1H), 8.61 (s, 1H), 8.26 (dd, *J* = 8.1, 6.1 Hz, 1H), 8.15 (s, 1H), 4.76 (t, *J* = 7.0 Hz, 2H), 3.47 (d, *J* = 5.8 Hz, 2H), 2.12 (p, *J* = 6.5 Hz, 2H). **^{13}C NMR** (100 MHz, DMSO-d_6) δ 162.8, 146.7, 144.9, 143.4, 133.6, 127.7, 59.2, 57.3, 33.1. **HRMS** *m*/*z* calculated for $C_9H_{13}N_2O_2$ $[M]^+$: 181.09715, found: 181.09715.

1-(Carbamoylmethyl)-3-carbamoylpyridinium bromide [CAS 1417623-14-3]

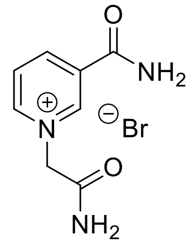

Nicotinamide (5 g, 40.9 mmol) and 2-bromoacetamide (6.2 g, 45 mmol) were mixed in acetonitrile (50 mL) and gently reflux overnight. Then the reaction mixture was cooled, the precipitate was filtered, washed with diethyl ether and dried. Recrystallization from aqueous ethanol afforded colorless crystals. **^{1}H NMR** (400 MHz, DMSO-d_6) δ 9.48 (t, *J* = 1.5 Hz, 1H), 9.14 (dd, *J* = 6.1, 1.3 Hz, 1H), 9.04 (dt, *J* = 8.1, 1.4 Hz, 1H), 8.62 (s, 1H), 8.29 (dd, *J* = 8.1, 6.1 Hz, 1H), 8.18 – 8.10 (m, 2H), 7.74 – 7.69 (m, 1H), 5.53 (s, 2H). **^{13}C NMR** (100 MHz, DMSO-d_6) δ 166.0, 162.8, 147.9, 146.3, 143.9, 133.1, 127.2, 109.6, 61.8. **HRMS** *m/z* calculated for $C_8H_{10}N_3O_2$ $[M]^+$: 180.07675, found: 180.0768.

1-(2-Carbamoylethyl)-3-carbamoylpyridinium bromide [CAS 41067-28-1]

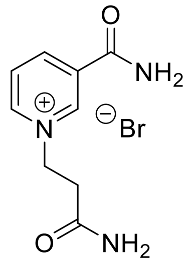

Nicotinamide (5 g, 40.9 mmol) and 3-bromopropionamide (12.4 g, 81.8 mmol) were mixed in acetonitrile (50 mL) and gently refluxed overnight. Then the reaction mixture was cooled, the precipitate was filtered, washed with Et_2O and dried. Recrystallization from aqueous ethanol resulted in colourless fine needle-shaped crystals. **^{1}H NMR** (400 MHz, DMSO-d_6) δ 9.64 (t, *J* = 1.4 Hz, 1H), 9.23 (dt, *J* = 6.2, 1.2 Hz, 1H), 9.00 (dt, *J* = 8.1, 1.4 Hz, 1H), 8.85 (s, 1H), 8.23 (dd, *J* = 8.1, 6.1 Hz, 1H), 8.15 (s, 1H), 7.64 (s, 1H), 7.05 (s, 1H), 4.85 (t, *J* = 6.6 Hz, 2H), 2.95 (t, *J* = 6.5 Hz, 2H). **^{13}C NMR** (101 MHz, DMSO-d_6) δ 170.7, 162.8, 147.0, 145.3, 143.7, 133.5, 127.4, 57.5, 35.3. **HRMS** *m/z* calculated for $C_9H_{12}N_3O_2$ $[M]^+$: 194.0924, found: 194.09251.

3. NMR spectra of the synthesised nicotinamide cofactor analogues

1-Benzyl-3-cyanopyridinium bromide [CAS 6516-53-6]

^{1}H NMR (400 MHz, DMSO-d_6)

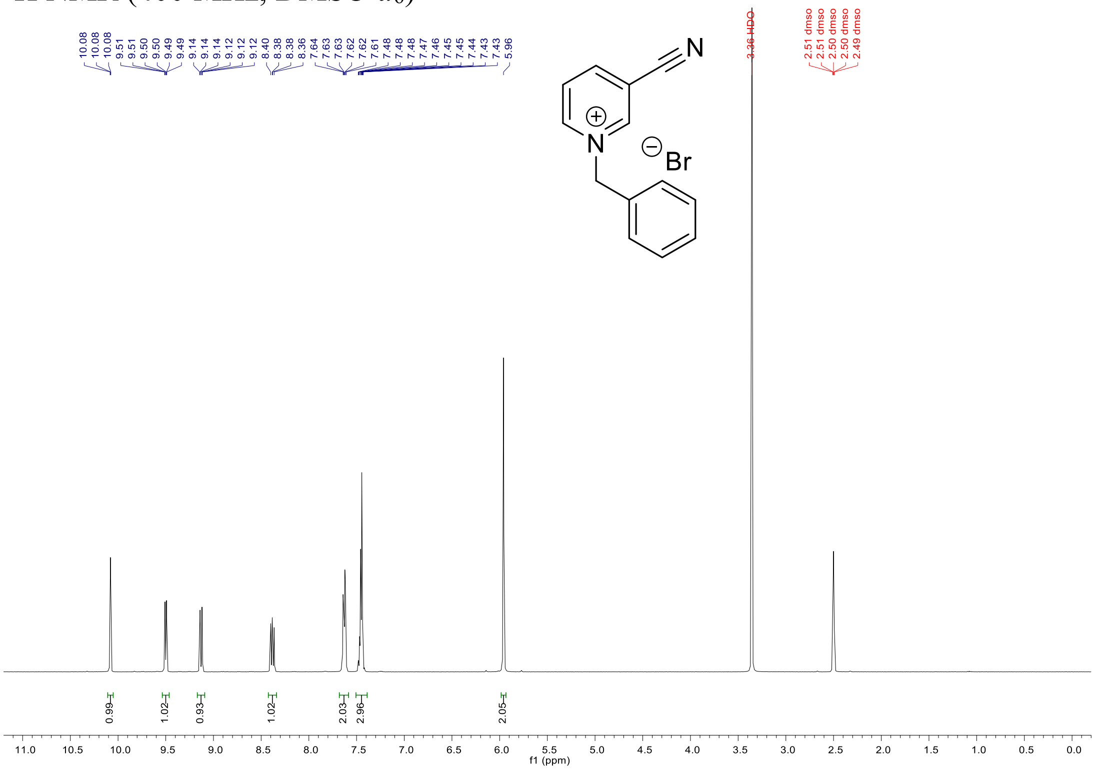

^{13}C NMR (100 MHz, DMSO-d_6)

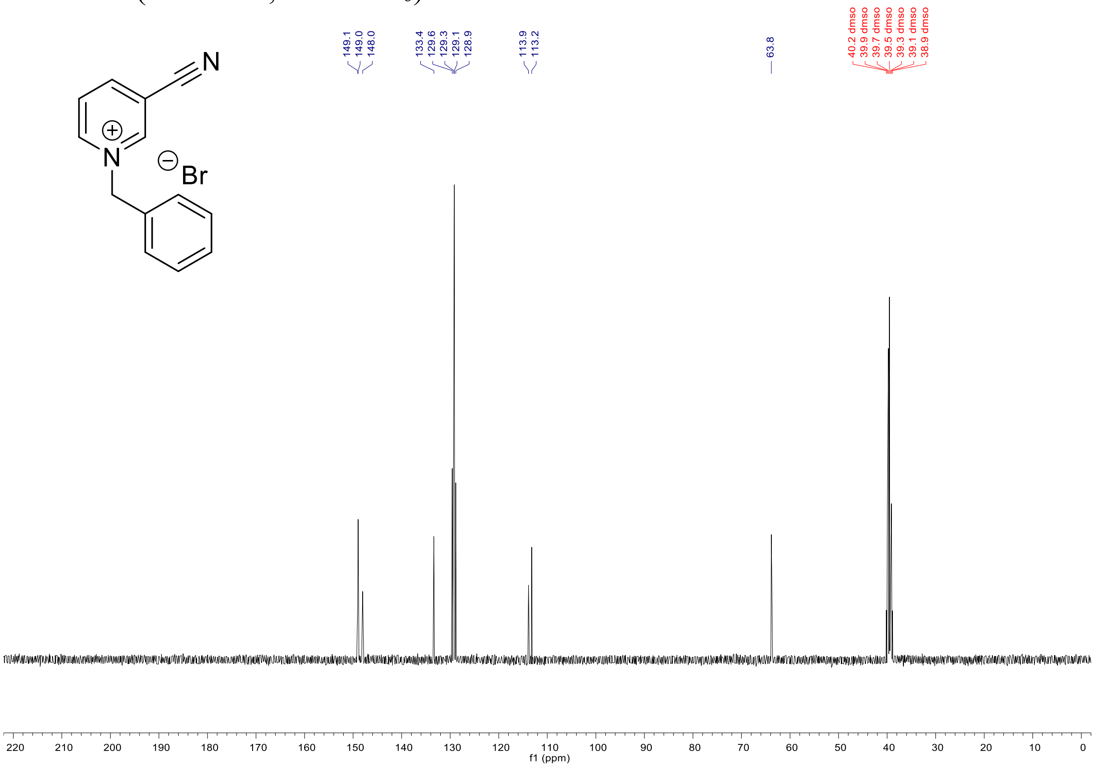

1-Methyl-3-carbamoylpyridinium iodide [CAS 6456-44-6]

^{1}H NMR (400 MHz, DMSO-d_6)

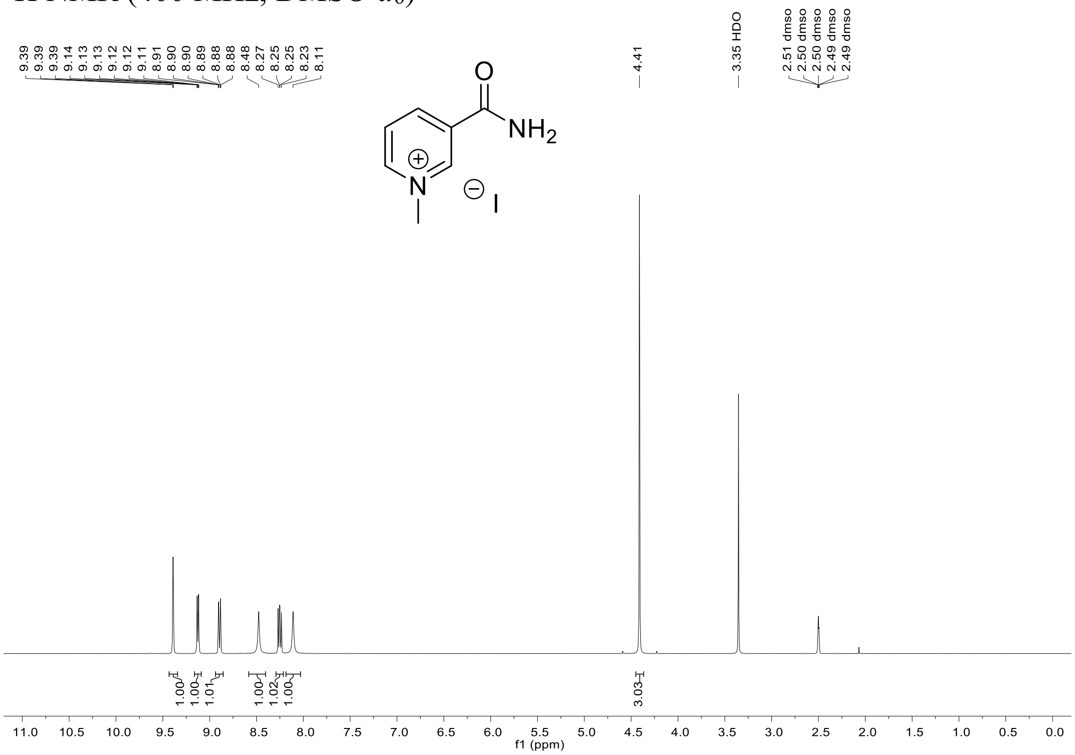

^{13}C NMR (100 MHz, DMSO-d_6)

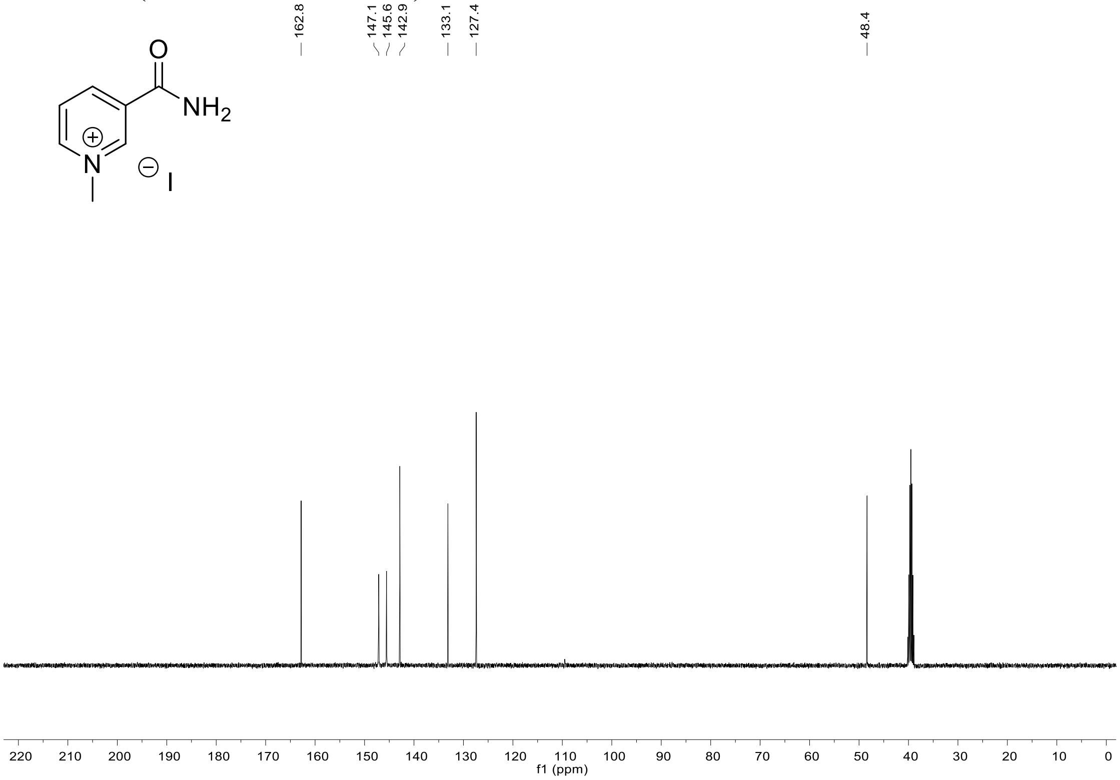

1-(2-Hydroxyethyl)-3-carbamoylpyridinium bromide [CAS 51527-80-1]

^{1}H NMR (400 MHz, DMSO-d_6)

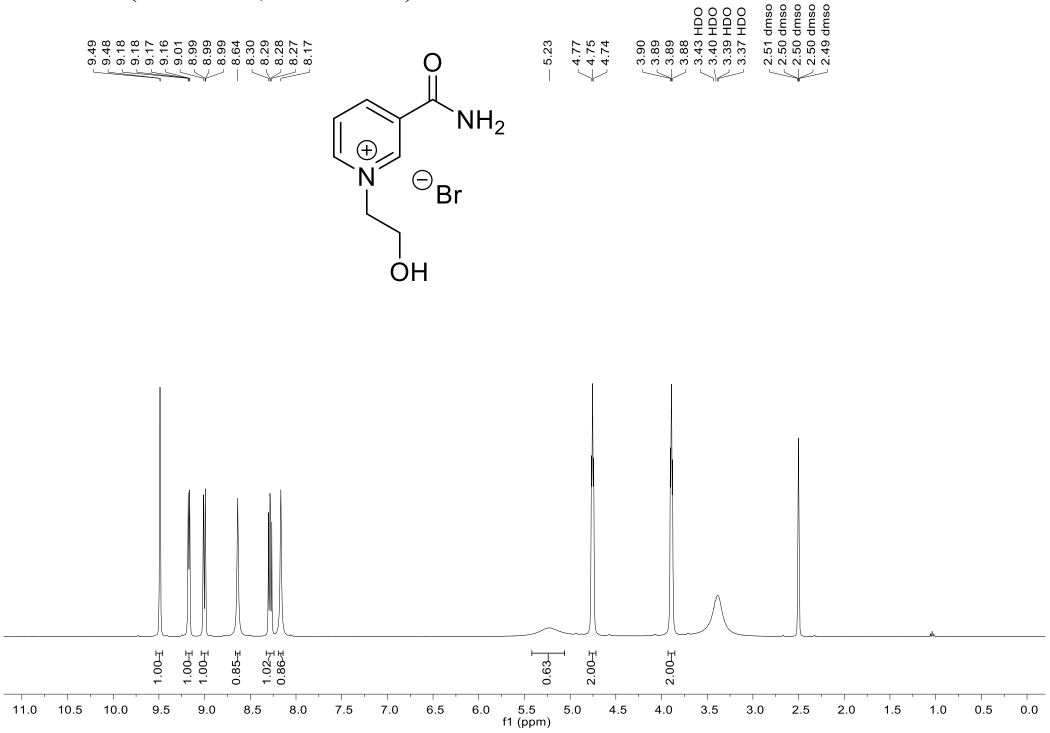

^{13}C NMR (100 MHz, DMSO-d_6)

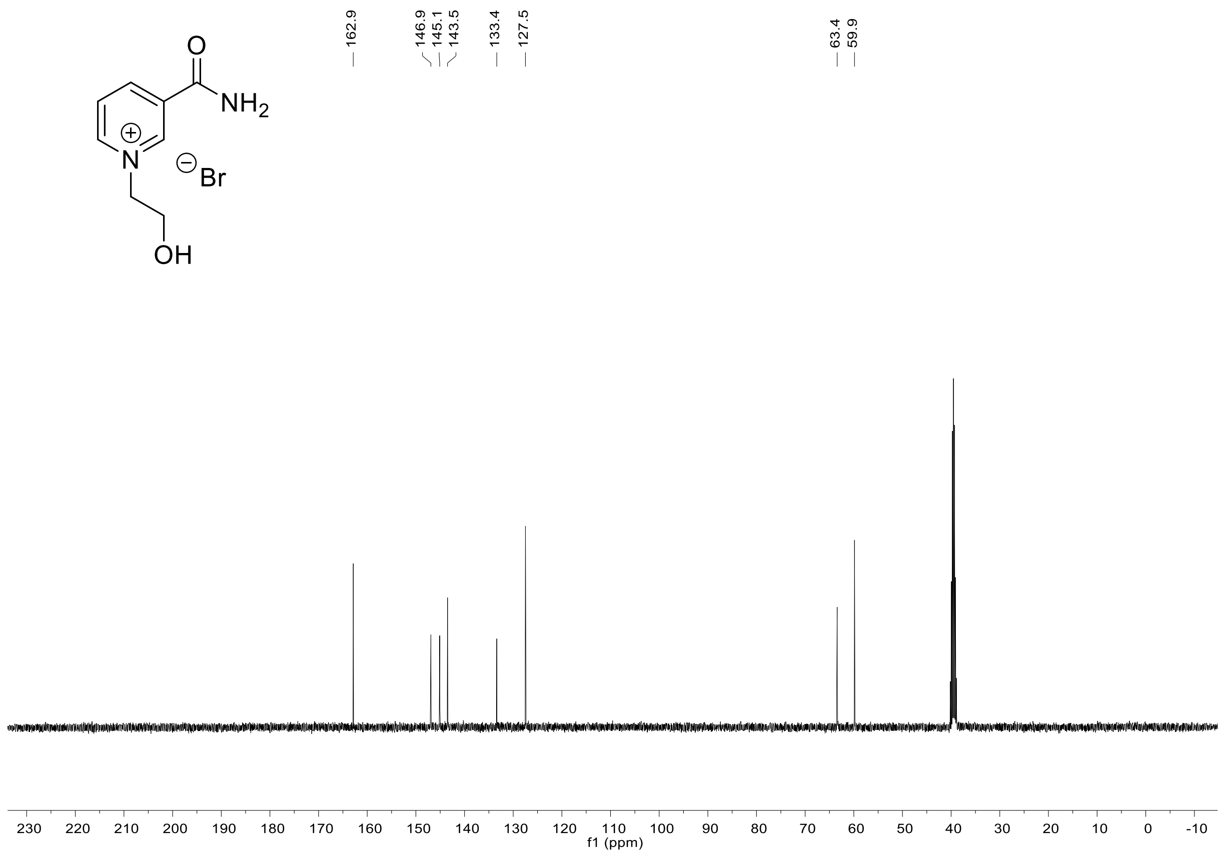

1-(3-Hydroxypropyl)-3-carbamoylpyridinium bromide [CAS 126298-92-8]

^{1}H NMR (400 MHz, DMSO-d_6)

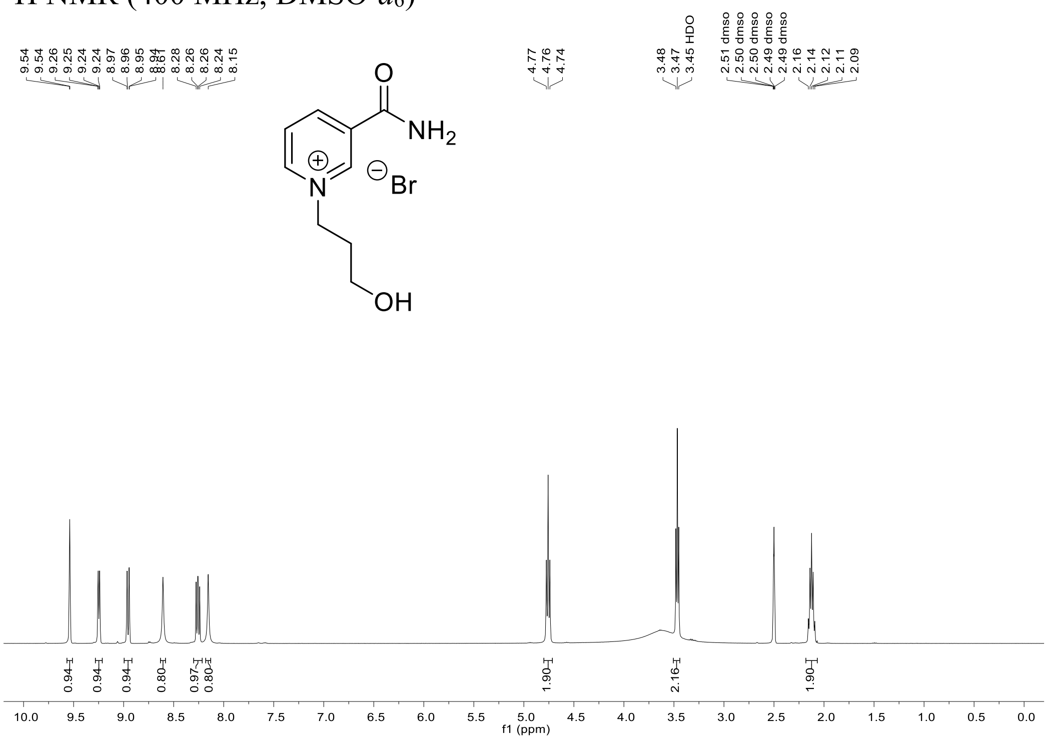

^{13}C NMR (100 MHz, DMSO-d_6)

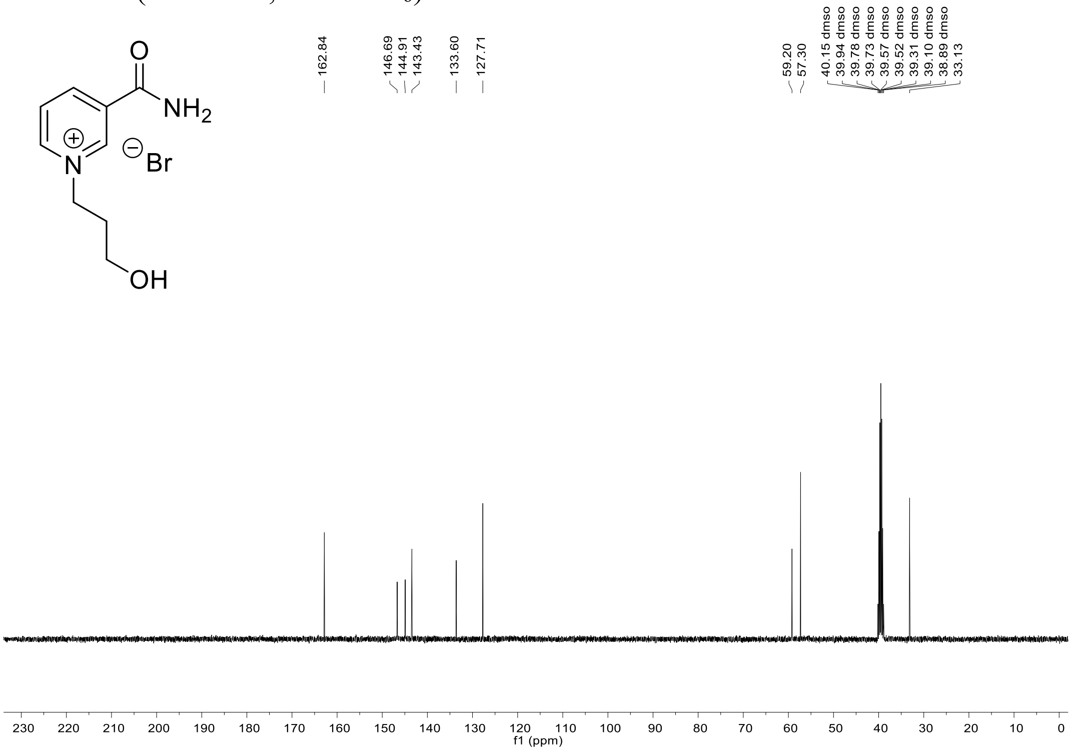

1-(Carbamoylmethyl)-3-carbamoylpyridinium bromide [CAS 1417623-14-3]

^{1}H NMR (400 MHz, DMSO-d_6)

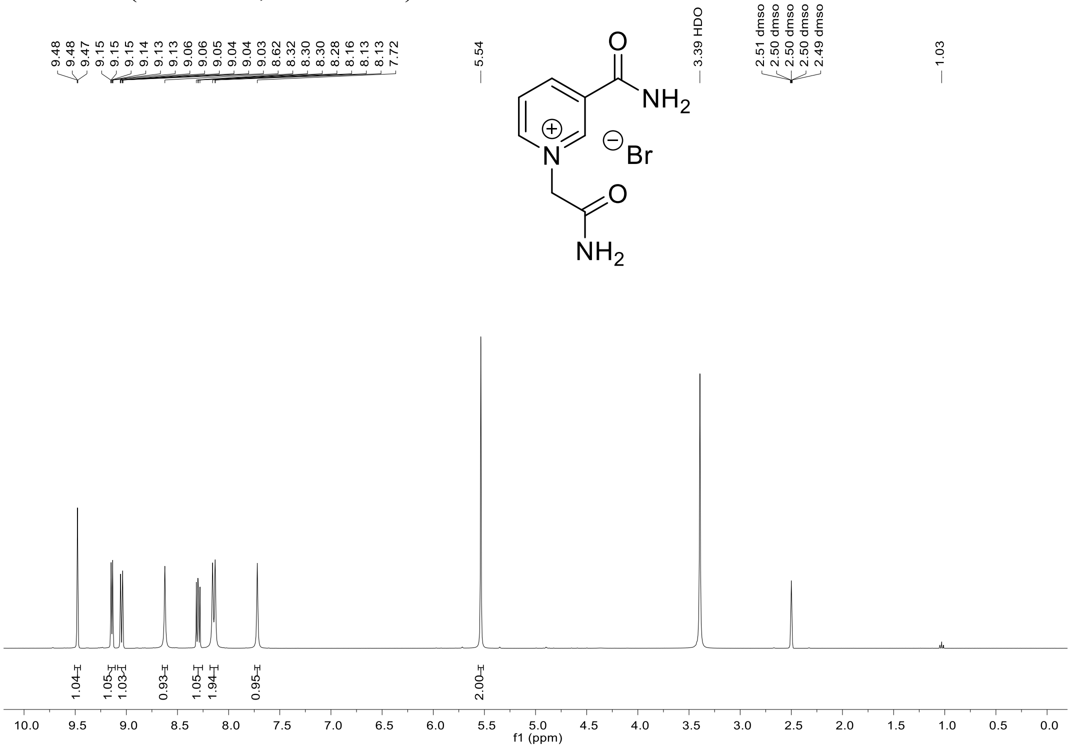

^{13}C NMR (100 MHz, DMSO-d_6)

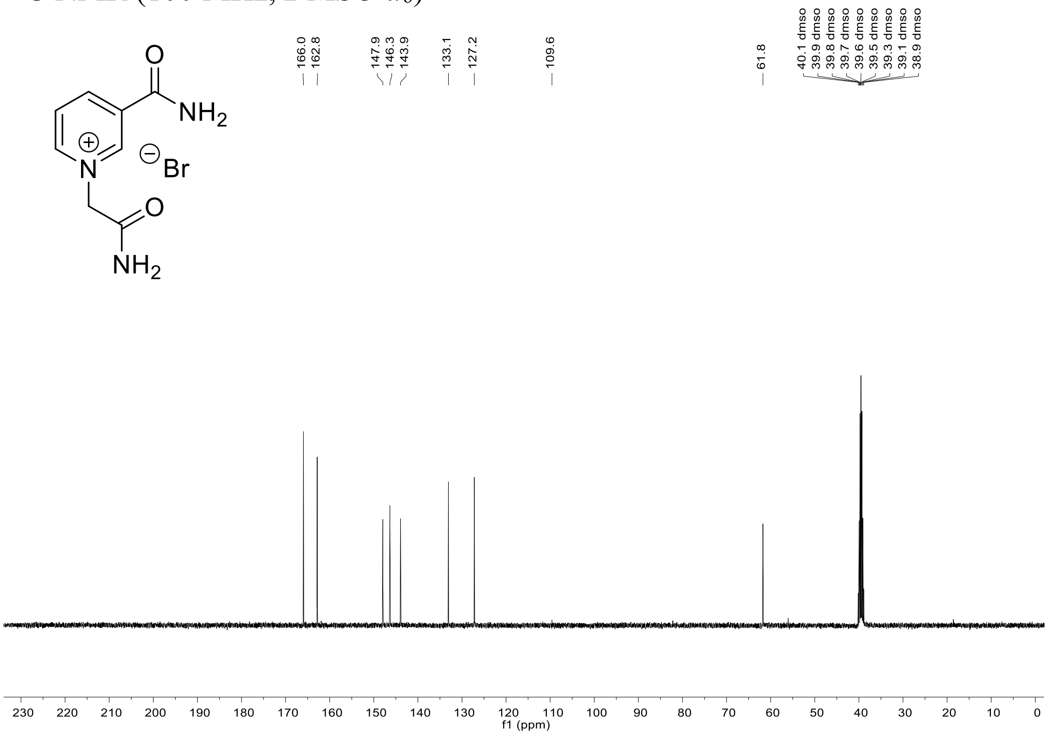

1-(2-Carbamoylethyl)-3-carbamoylpyridinium bromide [CAS 41067-28-1]

^{1}H NMR (400 MHz, DMSO-d_6)

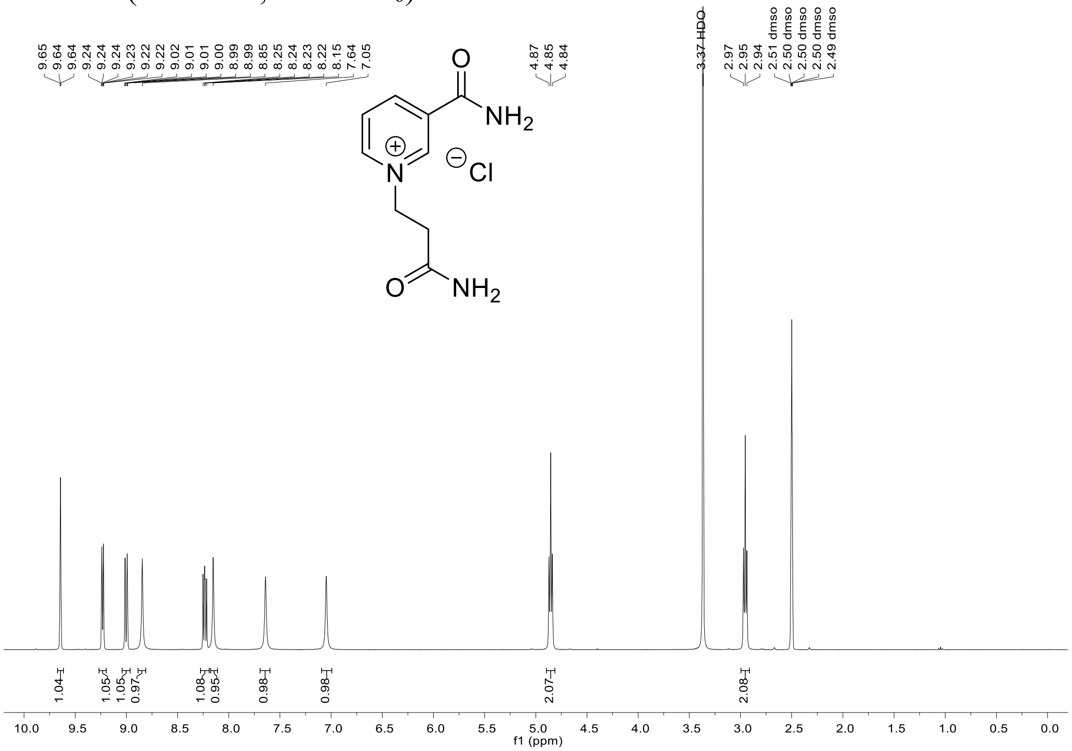

^{13}C NMR (100 MHz, DMSO-d_6)

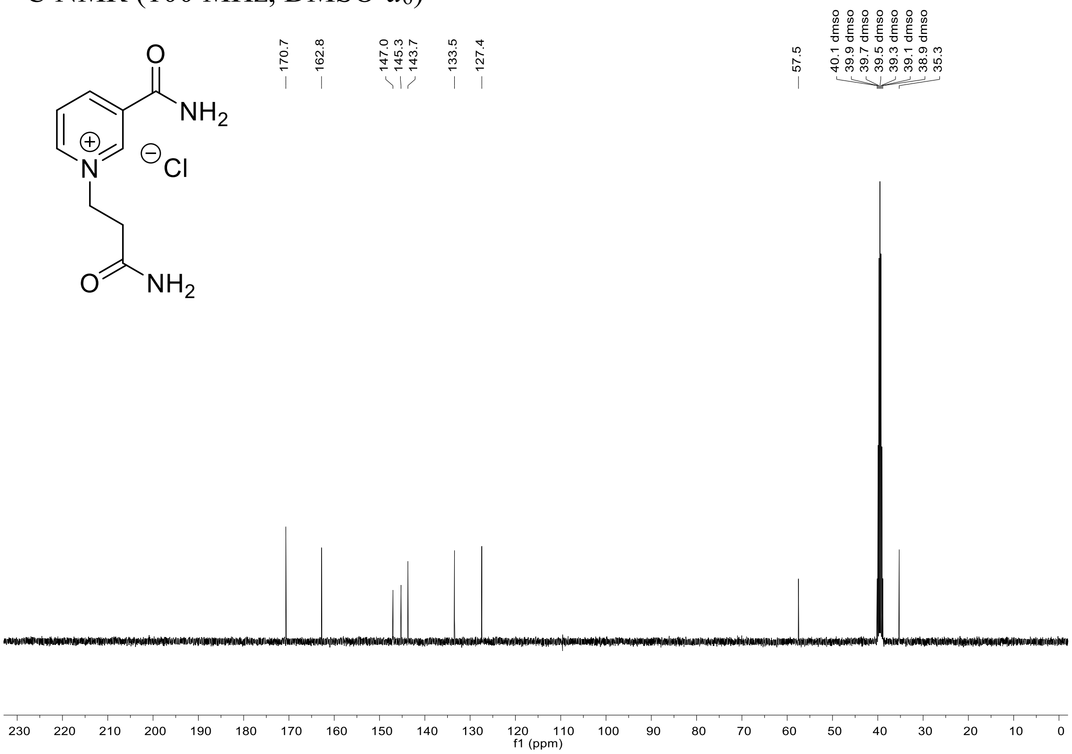

4. Supplementary Tables

Table S1. Data collection and structure refinement statistics.

**Data collection**	
Beamline	BESSY BL 14.1
Wavelength (Å)	0.9180
Temperature (K)	100
Crystal system / space group	monoclinic / $P2_1$
Cell dimensions a/b/c/β (Å,°)	90.6 / 124.4 / 92.7 / 103.7
Mosaicity (°)	0.10
Resolution range (Å)	47.08-1.92 (2.03-1.92)[1]
Reflections collected	869 895
Unique reflections	151 344
Multiplicity	5.7 (5.6)[1]
Completeness (%)	99.3 (98.4)[1]
$\langle I/\sigma I \rangle$	10.12 (2.16)[1]
$CC_{1/2}$	99.6 (79.4)[1]
R_{merge}[2]	0.124 (0.720)[1]
**Refinement**	
Working / test reflections	150 333 / 1 011
R / R_{free}[3]	0.1726 / 0.2175
Protein / Ado / water atoms	14 582 / 152 / 1 382
Na^+ ions / PEG molecules	4 / 1
$\langle B \rangle$ for protein / Ado / solvent ($Å^2$)	18.0 / 8.47 / 15.0
Rmsd from ideality for bonds (Å)	0.013
Ramachandran statistics: favored / allowed (%)	97.3 / 2.7
**PDB code**	6exi

[1]Values in parentheses correspond to the last resolution shell.
[2]$R_{merge} = \Sigma_{hkl} \Sigma_i |I_i(hkl) - \langle I(hkl) \rangle| / \Sigma_{hkl} \Sigma_i I_i(hkl)$, where $\langle I(hkl) \rangle$ is the average intensity of reflection hkl.
[3]$R = \Sigma_{hkl} | |F_o| - |F_c| | / \Sigma_{hkl} |F_o|$, where F_o and F_c are the observed and calculated structure factors, respectively. R_{free} is calculated analogously for the test reflections, which were randomly selected and excluded from the refinement.

Table S2. Michaelis-Menten kinetics at various inhibitor concentrations. The results are reported as averages of triplicates with their standard deviation.

**Concentration of NCB 10 [mM]**	**K_M [µM]**	**v_{max} [mM/min]**
0	33 ± 4	10.8 ± 0.3
5	35 ± 4	8.5 ± 0.2
15	47 ± 12	4.6 ± 0.3
20	42 ± 19	2.4 ± 0.3

5. References

[1] C. E. Paul, S. Gargiulo, D. J. Opperman, I. Lavandera, V. Gotor-Fernández, V. Gotor, A. Taglieber, I. W. C. E. Arends, F. Hollmann, *Org. Lett.* **2013**, *15*, 180-183.

[2] R. J. Knox, T. C. Jenkins, S. M. Hobbs, S. A. Chen, R. G. Melton, P. J. Burke, *Cancer Res.* **2000**, *60*, 4179-4186.
